# Explaining the entropy forming ability for carbides with the effective atomic size mismatch

**DOI:** 10.1038/s41598-024-57456-6

**Published:** 2024-03-26

**Authors:** Andreas Kretschmer, Paul Heinz Mayrhofer

**Affiliations:** Institute of Materials Science and Technology E308, TU Wien, Gumpendorferstrasse 7, 1060 Vienna, Austria

**Keywords:** High-entropy, Carbides, Hume-Rothery rules, Solid solutions, Phase stability, Atomistic models, Materials science, Ceramics

## Abstract

To quickly screen for single-phased multi-principal-element materials, a so-called entropy forming ability (EFA) parameter is sometimes used as a descriptor. The larger the EFA, the larger is the propensity to form a single-phase structure. We have investigated this EFA descriptor with atomic relaxations in special-quasi-random structures and discovered that the EFA correlates inversely with the lattice distortion. Large effective atomic size differences lead to multi-phase compounds, and little size differences to single-phase compounds. Instead of configurational entropy, we therefore demonstrate the applicability of the Hume-Rothery rules to phase stability of solid solutions even in compositionally complex ceramics.

## Introduction

The discovery of stable ceramic high-entropy materials is a daunting task due to the vast combinatorial space to search in. To speed up the exploration, we rely on descriptors that allow a rough estimate of the chance of success with low effort. One such example is the Hume-Rothery rules^1^, that provide a simple physical explanation for the formation of solid solutions in metals.

Of these Hume-Rothery rules, the size difference between constituting atoms, abbreviated with $\delta$, is most often used as descriptor to describe the propensity to form solid solutions in not only metallic, but also ceramic high-entropy materials. For example, Zhang et al^2^. used a data-driven approach to determine suitable elements for compositionally complex sulfides, while Jiang et al^3^. and Spiridigliozzi et al^4^.investigated the single-phase synthesis of complex oxides in perovskite and fluorite structure, respectively. Yan et al^5^. found that the formation of complex single-phase borides depends strongly on the deviation of the metal-B bond lengths. Tang et al^6^. used the bond strength in addition to the bond length to predict properties of carbides, nitrides, and carbonitrides, while Liu et al^7^.formulated a modified $\delta$ parameter which also takes the shear modulus mismatch into account. Zhou et al^8^. found that the most important features in predicting hardness and Young’s modulus in carbides with high configurational entropy are the valence electron concentration, the deviation of melting temperatures, and the fraction-weighted mean total energies, while atomic size differences plays a minor, but still significant role.

In contrast, the entropy forming ability (EFA)^9^ was formulated as a new descriptor for entropy stabilized compounds, and is sometimes interpreted as a driving force for local ordering, where a sufficiently large value indicates the propensity to form a single-phase solid solution due to configurational entropy. Compared to previous descriptors like the Hume-Rothery rules, the configurational entropy is claimed to be driving mechanism here. Intrigued by this descriptor, we investigated the same carbides as Sarker et al^9^. with ab initio calculations—implementing special quasi-random structures (SQS)—to analyze the local lattice distortion in order to develop a better understanding of the EFA.

## Methods

We performed all ab initio calculations using the Vienna Ab initio Simulation Package (VASP)^10,11^ with projector-augmented plane wave pseudo-potentials with generalized gradient approximated exchange-correlation functionals^12^. To analyze the local distortions in the structures, we used $2\times 2\times 2$ SQS supercells^13,14^, adapting our method established in^15^ for nitrides to the relevant 5-metal carbides in the phase space of Hf, Mo, Nb, Ta, Ti, V, W, and Zr. To improve the statistical ensemble, we have calculated 10 independent 64-atom cells per composition and use average values over these 10 cells. k-meshes^16^ and other details of the calculations can be found in the supplementary material. We quantify the local lattice distortion with a radial distribution function, in which the parameter $\sigma _1$ describes the distribution of bond lengths in the first coordination sphere (nearest neighbors),

1 $$\begin{aligned} rdf(r)=a_i\cdot e^{-\frac{(r-r_{0,i})^2}{2\sigma _i^2}}, \end{aligned}$$

so that a large effective atomic size mismatch leads to large values of $\sigma _1$, with $r_{0,i}$ as the mean bond length of the *i*th coordination sphere, $a_i$ as a fitting parameter for the distribution peak height, and $\sigma _i^2$ as the variance of the *i*th-neighbor bond length distribution. We contrast this parameter with the commonly used nominal deviation of the average atomic radius $\delta$,

2 $$\begin{aligned} \delta =\sqrt{\sum _{i=1}^{N}X_i\left( 1-\frac{r_i}{\overline{r}}\right) ^2}, \end{aligned}$$

where $X_i$ is the mole fraction of the *i*th component, $r_i$ the nearest neighbor C-metal bond length of the *i*th metal, and $\overline{r}$ the average nearest neighbor C-metal bond length of all metals present^17^. Since the actual atomic radii in ceramics can deviate from the radii tabulated for metals, we use the metal-carbon bond lengths of the respective binary face-centered cubic (fcc, NaCl prototype) cells instead of tabulated values to calculate $\delta$.

To complement and confirm our results, we have run more SQS calculations on multicomponent carbides in the same phase space with 2, 3, and 4 metals, and compared the resulting lattice distortion with EFA calculations, which we have prepared with the AFLOW-POCC^18^ module, which results in 2, 3, and 18 individual ordered structures for carbides with 2, 3, and 4 metals, respectively. Structure files and parameters for all calculations are listed in the supplementary materials.

## Results and discussion

**Figure 1:**
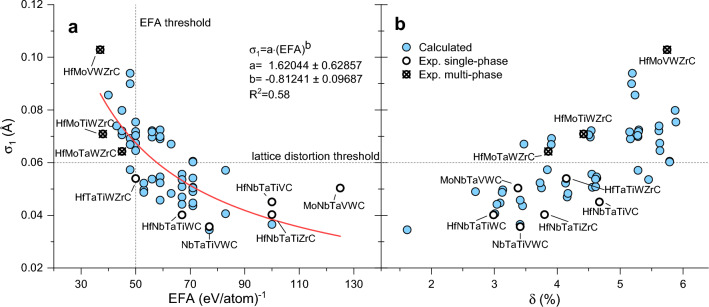
(**a**) The EFA shows a clear correlation with the local lattice distortion, quantified by the distribution of nearest neighbor bond lengths, $\sigma _1$. A power law fit (red line) illustrates the trend. The EFA data and experimental results (bold circles) are taken from^9^. (**b**) The nominal size variation $\delta$ correlates with $\sigma _1$, but is not precise enough to separate single- and multi-phase compositions its own.

We have mapped the correlation between $\sigma _1$ and the EFA of the 56 compounds studied in^9^ in Fig. 1**a**. The stated threshold for single-phase structures is at EFA= 50 (eV/atom)^-1^, which is shown as a dashed line. A power law fit (red line) illustrates that large lattice distortions occur only in compositions with small EFA values. Our data demonstrates that the EFA correlates with size effects of the constituting atoms, which has not been recognized before.

Sarker et al^9^. have argued that the formation of a single-phase (Mo,Nb,Ta,V,W)C is counter-intuitive with the argument, that two of the constituting metals, W, and Mo, prefer other structures and stoichiometric ratios. Our structural data helps to understand this oddity, showing that from the point of atomic size mismatch, the only moderate lattice distortion allows the formation of a single-phase material with these metals. This is especially true, if synthesis techniques such as spark plasma sintering or physical vapor deposition with their very fast cooling rates are used, allowing for kinetically-limited single-phase stabilization.

There is significant scattering in the data, causing the exemplary fitting function to display an $R^2$ score of only 0.58, but this fit is only intended to show the general trend. One source of this scattering we found to be in the EFA calculations, which are extremely sensitive to the convergence parameters. The total energies of the individual relaxed cells that we have calculated differ only by magnitudes in the meV range, so that slightly different—but high—stopping criteria for relaxation can lead to wildly different EFA scores. We therefore advise utmost caution when calculating this descriptor.

The other source of scattering is the fitting procedure for our $\sigma _1$ parameter. When the crystalline lattice shows only very little distortion—meaning the peak of the radial distribution function is very narrow—the fitted width of the Gaussian distribution of this narrow peak suffers from numerical inaccuracy, but when the distribution becomes broader, the fit becomes well defined. In effect this means that our $\sigma _1$ parameter cannot reliably quantify small differences in lattices with low distortion, but is effective at discerning lattices with little distortion from more distorted ones. Applying a similar method for defining a threshold for single-phase structures as Sarker et al^9^., we use roughly half the distance between the multi-phase and single-phase compositions, which are closest in their EFA values, (HfMoTaWZr)C and (HfTaTiWZr)C, to estimate an approximate threshold of $\sigma _1\approx$ 0.06Å, shown as a horizontal dashed line in Fig. 1, above which the lattice is too distorted to maintain a single phase. We must stress however, that just like the EFA threshold this is a purely empirical estimation based on rather little data, and thus may not be very reliable.

A simpler approach to the lattice distortion is the nominal size mismatch $\delta$, which correlates with $\sigma _1$, see Fig. 1**b**. Despite using metal-carbon bond lengths as size parameter, the $\delta$ parameter fails to separate single- and multi-phase compositions. The underlying principle of the Hume-Rothery rules is still valid, but since atomic radii are influenced by local charge transfer^19^, atomic radii are ill defined in complex solid solutions. Therefore, the simple $\delta$ parameter fails to capture the chemical complexity involved. Instead, the local relaxations in our SQS cells provide a better guide to real distortions of the lattice and can be used to gauge the single-phase stability.

**Figure 2:**
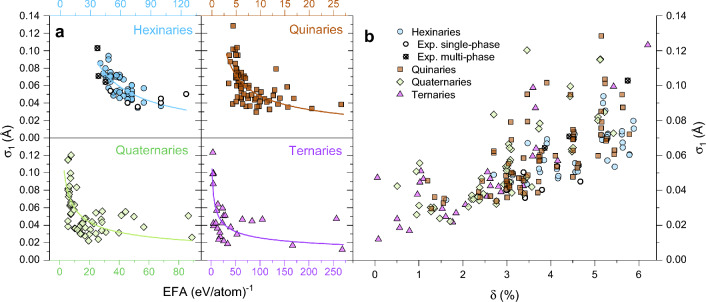
(**a**) The different multinary level alloys all show the same correlation between EFA and $\sigma _1$. The lines represent power law fits as in Fig. 1a for every multinary level. Note, that the scaling of EFA is different for every multinary level. (**b**) The correlation between $\delta$ and $\sigma _1$ is also apparent at all multinary levels. Note, that at large values of $\delta$, the lattice distortion $\sigma _1$ is always significant.

To confirm our finding, we conducted further calculations of the EFA and $\sigma _1$ on the multinary carbides with 2, 3, and 4 of the same metals. The correlation between the additional datasets is depicted in Fig. 2a for all multinary levels, demonstrating trends identical to the one shown in Fig. 1a. A power law fit for every multinary level is inserted as guide line with the corresponding color. Please note, that the scaling of the EFA is different in this figure for every multinary level. Thus, an EFA of 50 (eV/atom)^-1^ is not the general threshold to form a single-phase material.

The relationship between $\sigma _1$ and $\delta$ is shown for all considered compositions in Fig. 2b. Large variations in $\sigma _1$ for a given $\delta$ demonstrate only a moderate reliability of the $\delta$ parameter to gauge the actual lattice distortion, local lattice distortions from ab initio calculations were also shown to be useful to estimate the ductility of alloys, whereas $\delta$ is not accurate enough^20^. As we would expect, we see a total absence of small lattice distortion in compounds with large differences of atomic radii, which serves as a sanity check that our method works correctly.

**Figure 3:**
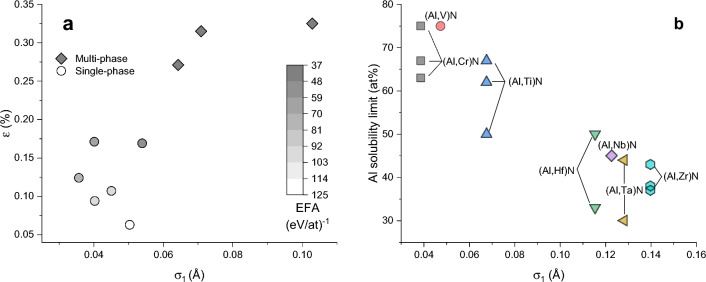
(**a**) The calculated lattice distortion $\sigma _1$ correlates well with the XRD measured interplanar spacing $\epsilon$ of the nine experimentally investigated carbides. The grayscale filling denotes the corresponding EFA values. $\epsilon$ and EFA values are taken from^9^. (**b**) A second application of $\sigma _1$ is the prediction of the maximum solubility limit of Al on the metal sublattice of metastable, physical vapor deposited nitrides in B1 structure, using the data from^21–34^.

Additionally, with the different sizes of the constituting atoms—and thereby roughened crystal planes—we can easily explain the experimental findings presented in^9^ over the whole data range. While the interplanar spacing $\epsilon$, taken as the X-ray diffraction-measured reflex width in^9^, inversely correlates with the EFA for both single- and multi-phased materials, $\epsilon$ also correlates strongly with the calculated lattice distortion $\sigma _1$, see Fig. 3a, confirming our calculations.

We can strengthen our argument on size effects by applying the $\sigma _1$ descriptor to predict the stability of solid solutions for other materials as well. Using the $\sigma _1$ values of the isostructural nitrides, which we have calculated with the same method in^15^, we can correlate the lattice distortion in the nitrides with the maximum solubility limit of Al in physical vapor deposited (PVD) ($\textrm{TM}_{x}\textrm{Al}_{1-x})\textrm{N}$ coatings, see Fig. 3b. These nitrides are only metastable in the B1 structure, since Al strongly favors the hexagonal B4 structure, but the structure can be stabilized kinetically through high-energy bombardment and high cooling rates, which are characteristic for PVD. The extent of this stabilization depends strongly on the transition metal. Extensive studies have been performed on several of these nitrides, most of all (Al,Ti)N^21–23^ and (Al,Cr)N^24–26^, while (Al,V)N^27^, (Al,Hf)N^21,28^, (Al,Nb)N^29^, (Al,Ta)N^30,31^, and (Al,Zr)N^21,32,33^ are less explored. Optimizing the deposition parameters influences the maximum solubility limit significantly, but we see a clear trend that the metal pairs with similar atom sizes can stabilize more Al in the B1 structure and this is well reflected in the lattice distortion, parameterized by $\sigma _1$. In these simple cases, the nominal $\delta$ parameter would also be sufficient to derive the same conclusion. The analysis of more complex nitrides would be a more interesting exercise, but the Al-solubility limits are not yet explored in these.

## Conclusion

The EFA as a descriptor was defined in^9^ to map the energy cost of accessing metastable configurations. The authors interpreted the agency behind their formed solid solutions as entropy stabilization. Here, we can give a more basic explanation of their finding, namely that this energy cost is directly related to the mismatch in atomic sizes and the resulting lattice distortion. The underlying Hume-Rothery rules for solid solution therefore remain central for the stability of solid solutions also in high-entropy compounds.

Our radial distribution analysis of bond lengths in relaxed special quasi-random structures allows us to capture the atomic size dependent lattice distortion in an easily interpretable way. Since charge transfer in alloys influences the effective atomic sizes, we require this ab initio treatment to demonstrate the relationship between the magnitude of the EFA descriptor and the effective atomic size deviation, while the simple $\delta$ parameter is not precise enough. We can therefore complement the Hume-Rothery rule regarding the size mismatch, demanding instead of similar atomic radii, a sufficiently small effective lattice distortion. Based on the little available data, we quantify the threshold for the present carbides as $\sigma _1\approx$ 0.06 Å, below which single phase solid solution carbides can form.

### Supplementary Information

Supplementary Information.

## Data Availability

A part of the raw data can be found in the material. More data supporting the findings of this study is available from the corresponding author upon request.
