# Supplementary material for: Explaining the entropy forming ability for carbides with the effective atomic size mismatch
Source: Sci Rep. 2024 Mar 26;14:7210. doi: 10.1038/s41598-024-57456-6 (PMC10966041; doi:10.1038/s41598-024-57456-6)
Supplement: Supplementary file 1 — Supplementary Information. [file 41598_2024_57456_MOESM1_ESM.pdf]

Explaining the Entropy Forming Ability with the atomic size mismatch

Supplementary Materials

Andreas Kretschmer*, Paul Heinz Mayrhofer

* Corresponding Author: andreas.kretschmer@tuwien.ac.at

TU Wien, Institute of Materials Science and Technology

Gumpendorferstrasse 7, 1060 Vienna, Austria

Contents

1 EFA calculations **1**

1.1 Ternaries 1

1.2 Quaternaries 2

1.3 Quinaries 2

2 SQS calculations **10**

2.1 Binaries 10

2.2 Ternaries 10

2.3 Quaternaries 12

2.4 Quinaries 16

2.5 Hexinaries 17

3 Plot data **30**

References **35**

1 EFA calculations

All EFA calculations have been performed with a cutoff energy of 600 eV, the stopping criterion for ionic relaxation was set to 10^{-6} eV. The Γ-centered Monkhorst-Pack k-mesh [1] was automatically generated with a subdivision length R_k set to 50, the explicit values are listed in Supplementary Table 1. The following structures for the EFA calculations have been generated by the AFLOW-POCC [2] algorithm. The structures are given in the POSCAR format. All possible combinations of Hf, Mo, Nb, Ta, Ti, V, W, and Zr have been placed on the metal sites E1,...,E4 in line 6 of every structure file.

1.1 Ternaries

```
Ternary_1 HNF(n=2,1/2)=[1 0 0; 0 1 0; 0 0 2] site_config(1/2)=[0 0; 1 2] DG=2
1.000000
0.00000000000000   2.34443833412019   2.34443833412019
2.34443833412019   0.00000000000000   2.34443833412019
4.68887666824038   4.68887666824038   0.00000000000000
E1 E2 C
1 1 2
Direct(4) [A2B1C1]
0.50000000000000   0.50000000000000   0.25000000000000
0.50000000000000   0.50000000000000   0.75000000000000
0.00000000000000   0.00000000000000   0.00000000000000
0.00000000000000   0.00000000000000   0.50000000000000
```

```
Ternary_2 HNF(n=2,2/2)=[1 0 0; 0 1 1; 0 0 2] site_config(1/2)=[0 0; 1 2] DG=2
1.000000
0.00000000000000   2.34443833412019   2.34443833412019
4.68887666824038   2.34443833412019   2.34443833412019
4.68887666824038   4.68887666824038   0.00000000000000
E1 E2 C
1 1 2
Direct(4) [A2B1C1]
0.50000000000000   0.50000000000000   0.00000000000000
0.50000000000000   0.50000000000000   0.50000000000000
0.00000000000000   0.00000000000000   0.00000000000000
0.00000000000000   0.00000000000000   0.50000000000000
```

1.2 Quaternaries

```
Quaternary_1 HNF(n=3,1/3)=[1 0 0; 0 1 0; 0 0 3] site_config(1/6)=[0 0 0; 1 2 3] DG=6
1.000000
0.00000000000000   2.31295150667741   2.31295150667741
2.31295150667741   0.00000000000000   2.31295150667741
6.93885452003224   6.93885452003224   0.00000000000000
E1 E2 E3 C
1 1 1 3
Direct(6) [A3B1C1D1]
0.50000000000000   0.50000000000000   0.16666666666667
0.50000000000000   0.50000000000000   0.50000000000000
0.50000000000000   0.50000000000000   0.83333333333333
0.00000000000000   0.00000000000000   0.00000000000000
0.00000000000000   0.00000000000000   0.33333333333333
0.00000000000000   0.00000000000000   0.66666666666667

Quaternary_2 HNF(n=3,2/3)=[1 0 0; 0 1 1; 0 0 3] site_config(1/6)=[0 0 0; 1 2 3] DG=6
1.000000
0.00000000000000   2.31295150667741   2.31295150667741
4.62590301335482   2.31295150667741   2.31295150667741
6.93885452003224   6.93885452003224   0.00000000000000
E1 E2 E3 C
1 1 1 3
Direct(6) [A3B1C1D1]
0.50000000000000   0.50000000000000   0.00000000000000
0.50000000000000   0.50000000000000   0.33333333333333
0.50000000000000   0.50000000000000   0.66666666666667
0.00000000000000   0.00000000000000   0.00000000000000
0.00000000000000   0.00000000000000   0.33333333333333
0.00000000000000   0.00000000000000   0.66666666666667

Quaternary_3 HNF(n=3,3/3)=[1 0 0; 0 1 2; 0 0 3] site_config(1/6)=[0 0 0; 1 2 3] DG=6
1.000000
0.00000000000000   2.31295150667741   2.31295150667741
6.93885452003224   4.62590301335482   2.31295150667741
6.93885452003224   6.93885452003224   0.00000000000000
E1 E2 E3 C
1 1 1 3
Direct(6) [A3B1C1D1]
0.50000000000000   0.50000000000000   0.16666666666667
0.50000000000000   0.50000000000000   0.50000000000000
0.50000000000000   0.50000000000000   0.83333333333333
0.00000000000000   0.00000000000000   0.00000000000000
0.00000000000000   0.00000000000000   0.33333333333333
0.00000000000000   0.00000000000000   0.66666666666667
```

1.3 Quinaries

```
Quin_1 H0C0 HNF(n=4,1/7)=[1 0 0; 0 1 0; 0 0 4] site_config(1/24)=[0 0 0 0; 1 2 3 4] DG=8
1.000000
0.00000000000000   2.26569844629123   2.26569844629123
2.26569844629123   0.00000000000000   2.26569844629123
9.06279378516493   9.06279378516493   0.00000000000000
```

```
E1 E2 E3 E4 C
1 1 1 1 4
Direct(8) [A4B1C1D1E1]
0.50000000000000   0.50000000000000   0.12500000000000
0.50000000000000   0.50000000000000   0.37500000000000
0.50000000000000   0.50000000000000   0.62500000000000
0.50000000000000   0.50000000000000   0.87500000000000
0.00000000000000   0.00000000000000   0.00000000000000
0.00000000000000   0.00000000000000   0.25000000000000
0.00000000000000   0.00000000000000   0.50000000000000
0.00000000000000   0.00000000000000   0.75000000000000

Quin_10 H3C0 HNF(n=4,4/7)=[1 0 0; 0 1 3; 0 0 4] site_config(1/24)=[0 0 0 0; 1 2 3 4] DG=8
1.000000
0.00000000000000   2.26569844629123   2.26569844629123
9.06279378516493   6.79709533887370   2.26569844629123
9.06279378516493   9.06279378516493   0.00000000000000
E1 E2 E3 E4 C
1 1 1 1 4
Direct(8) [A4B1C1D1E1]
0.50000000000000   0.50000000000000   0.00000000000000  E1
0.50000000000000   0.50000000000000   0.25000000000000  E2
0.50000000000000   0.50000000000000   0.50000000000000  E3
0.50000000000000   0.50000000000000   0.75000000000000  E4
0.00000000000000   0.00000000000000   0.00000000000000  C
0.00000000000000   0.00000000000000   0.25000000000000  C
0.00000000000000   0.00000000000000   0.50000000000000  C
0.00000000000000   0.00000000000000   0.75000000000000  C

Quin_11 H3C1 HNF(n=4,4/7)=[1 0 0; 0 1 3; 0 0 4] site_config(2/24)=[0 0 0 0; 1 2 4 3] DG=8
1.000000
0.00000000000000   2.26569844629123   2.26569844629123
9.06279378516493   6.79709533887370   2.26569844629123
9.06279378516493   9.06279378516493   0.00000000000000
E1 E2 E3 E4 C
1 1 1 1 4
Direct(8) [A4B1C1D1E1]
0.50000000000000   0.50000000000000   0.00000000000000  E1
0.50000000000000   0.50000000000000   0.25000000000000  E2
0.50000000000000   0.50000000000000   0.75000000000000  E3
0.50000000000000   0.50000000000000   0.50000000000000  E4
0.00000000000000   0.00000000000000   0.00000000000000  C
0.00000000000000   0.00000000000000   0.25000000000000  C
0.00000000000000   0.00000000000000   0.50000000000000  C
0.00000000000000   0.00000000000000   0.75000000000000  C

Quin_12 H3C2 HNF(n=4,4/7)=[1 0 0; 0 1 3; 0 0 4] site_config(3/24)=[0 0 0 0; 1 3 2 4] DG=8
1.000000
0.00000000000000   2.26569844629123   2.26569844629123
9.06279378516493   6.79709533887370   2.26569844629123
9.06279378516493   9.06279378516493   0.00000000000000
E1 E2 E3 E4 C
1 1 1 1 4
Direct(8) [A4B1C1D1E1]
0.50000000000000   0.50000000000000   0.00000000000000  E1
0.50000000000000   0.50000000000000   0.50000000000000  E2
0.50000000000000   0.50000000000000   0.25000000000000  E3
```

```
0.50000000000000   0.50000000000000   0.75000000000000  E4
0.00000000000000   0.00000000000000   0.00000000000000  C
0.00000000000000   0.00000000000000   0.25000000000000  C
0.00000000000000   0.00000000000000   0.50000000000000  C
0.00000000000000   0.00000000000000   0.75000000000000  C

Quin_13 H4C0 HNF(n=4,5/7)=[1 0 1; 0 1 2; 0 0 4] site_config(1/24)=[0 0 0 0; 1 2 3 4] DG=16
1.000000
2.26569844629123   4.53139689258246   2.26569844629123
6.79709533887370   4.53139689258246   2.26569844629123
9.06279378516493   9.06279378516493   0.00000000000000
E1 E2 E3 E4 C
1 1 1 1 4
Direct(8) [A4B1C1D1E1]
0.50000000000000   0.50000000000000   0.00000000000000  E1
0.50000000000000   0.50000000000000   0.25000000000000  E2
0.50000000000000   0.50000000000000   0.50000000000000  E3
0.50000000000000   0.50000000000000   0.75000000000000  E4
0.00000000000000   0.00000000000000   0.00000000000000  C
0.00000000000000   0.00000000000000   0.25000000000000  C
0.00000000000000   0.00000000000000   0.50000000000000  C
0.00000000000000   0.00000000000000   0.75000000000000  C

Quin_14 H4C2 HNF(n=4,5/7)=[1 0 1; 0 1 2; 0 0 4] site_config(3/24)=[0 0 0 0; 1 3 2 4] DG=8
1.000000
2.26569844629123   4.53139689258246   2.26569844629123
6.79709533887370   4.53139689258246   2.26569844629123
9.06279378516493   9.06279378516493   0.00000000000000
E1 E2 E3 E4 C
1 1 1 1 4
Direct(8) [A4B1C1D1E1]
0.50000000000000   0.50000000000000   0.00000000000000  E1
0.50000000000000   0.50000000000000   0.50000000000000  E2
0.50000000000000   0.50000000000000   0.25000000000000  E3
0.50000000000000   0.50000000000000   0.75000000000000  E4
0.00000000000000   0.00000000000000   0.00000000000000  C
0.00000000000000   0.00000000000000   0.25000000000000  C
0.00000000000000   0.00000000000000   0.50000000000000  C
0.00000000000000   0.00000000000000   0.75000000000000  C

Quin_15 H5C0 HNF(n=4,6/7)=[1 0 0; 0 2 0; 0 0 2] site_config(1/24)=[0 0 0 0; 1 2 3 4] DG=8
1.000000
0.00000000000000   2.26569844629123   2.26569844629123
4.53139689258246   0.00000000000000   4.53139689258246
4.53139689258246   4.53139689258246   0.00000000000000
E1 E2 E3 E4 C
1 1 1 1 4
Direct(8) [A4B1C1D1E1]
0.50000000000000   0.25000000000000   0.25000000000000  E1
0.50000000000000   0.25000000000000   0.75000000000000  E2
0.50000000000000   0.75000000000000   0.25000000000000  E3
0.50000000000000   0.75000000000000   0.75000000000000  E4
0.00000000000000   0.00000000000000   0.00000000000000  C
0.00000000000000   0.00000000000000   0.50000000000000  C
0.00000000000000   0.50000000000000   0.00000000000000  C
0.00000000000000   0.50000000000000   0.50000000000000  C
```

```
Quin_16 H5C1 HNF(n=4,6/7)=[1 0 0; 0 2 0; 0 0 2] site_config(2/24)=[0 0 0 0; 1 2 4 3] DG=8
1.000000
0.00000000000000   2.26569844629123   2.26569844629123
4.53139689258246   0.00000000000000   4.53139689258246
4.53139689258246   4.53139689258246   0.00000000000000
E1 E2 E3 E4 C
1 1 1 1 4
Direct(8) [A4B1C1D1E1]
0.50000000000000   0.25000000000000   0.25000000000000  E1
0.50000000000000   0.25000000000000   0.75000000000000  E2
0.50000000000000   0.75000000000000   0.75000000000000  E3
0.50000000000000   0.75000000000000   0.25000000000000  E4
0.00000000000000   0.00000000000000   0.00000000000000  C
0.00000000000000   0.00000000000000   0.50000000000000  C
0.00000000000000   0.50000000000000   0.00000000000000  C
0.00000000000000   0.50000000000000   0.50000000000000  C

Quin_17 H5C3 HNF(n=4,6/7)=[1 0 0; 0 2 0; 0 0 2] site_config(4/24)=[0 0 0 0; 1 3 4 2] DG=8
1.000000
0.00000000000000   2.26569844629123   2.26569844629123
4.53139689258246   0.00000000000000   4.53139689258246
4.53139689258246   4.53139689258246   0.00000000000000
E1 E2 E3 E4 C
1 1 1 1 4
Direct(8) [A4B1C1D1E1]
0.50000000000000   0.25000000000000   0.25000000000000  E1
0.50000000000000   0.75000000000000   0.75000000000000  E2
0.50000000000000   0.25000000000000   0.75000000000000  E3
0.50000000000000   0.75000000000000   0.25000000000000  E4
0.00000000000000   0.00000000000000   0.00000000000000  C
0.00000000000000   0.00000000000000   0.50000000000000  C
0.00000000000000   0.50000000000000   0.00000000000000  C
0.00000000000000   0.50000000000000   0.50000000000000  C

Quin_18 H6C0 HNF(n=4,7/7)=[1 1 1; 0 2 0; 0 0 2] site_config(1/24)=[0 0 0 0; 1 2 3 4] DG=24
1.000000
4.53139689258246   4.53139689258246   4.53139689258246
4.53139689258246   0.00000000000000   4.53139689258246
4.53139689258246   4.53139689258246   0.00000000000000
E1 E2 E3 E4 C
1 1 1 1 4
Direct(8) [A4B1C1D1E1]
0.50000000000000   0.00000000000000   0.00000000000000  E1
0.50000000000000   0.00000000000000   0.50000000000000  E2
0.50000000000000   0.50000000000000   0.00000000000000  E3
0.50000000000000   0.50000000000000   0.50000000000000  E4
0.00000000000000   0.00000000000000   0.00000000000000  C
0.00000000000000   0.00000000000000   0.50000000000000  C
0.00000000000000   0.50000000000000   0.00000000000000  C
0.00000000000000   0.50000000000000   0.50000000000000  C

Quin_2 H0C1 HNF(n=4,1/7)=[1 0 0; 0 1 0; 0 0 4] site_config(2/24)=[0 0 0 0; 1 2 4 3] DG=8
1.000000
0.00000000000000   2.26569844629123   2.26569844629123
2.26569844629123   0.00000000000000   2.26569844629123
9.06279378516493   9.06279378516493   0.00000000000000
E1 E2 E3 E4 C
```

```
1 1 1 1 4
Direct(8) [A4B1C1D1E1]
0.50000000000000   0.50000000000000   0.12500000000000
0.50000000000000   0.50000000000000   0.37500000000000
0.50000000000000   0.50000000000000   0.87500000000000
0.50000000000000   0.50000000000000   0.62500000000000
0.00000000000000   0.00000000000000   0.00000000000000
0.00000000000000   0.00000000000000   0.25000000000000
0.00000000000000   0.00000000000000   0.50000000000000
0.00000000000000   0.00000000000000   0.75000000000000

Quin_3 H0C2 HNF(n=4,1/7)=[1 0 0; 0 1 0; 0 0 4] site_config(3/24)=[0 0 0 0; 1 3 2 4] DG=8
1.000000
0.00000000000000   2.26569844629123   2.26569844629123
2.26569844629123   0.00000000000000   2.26569844629123
9.06279378516493   9.06279378516493   0.00000000000000
E1 E2 E3 E4 C
1 1 1 1 4
Direct(8) [A4B1C1D1E1]
0.50000000000000   0.50000000000000   0.12500000000000  E1
0.50000000000000   0.50000000000000   0.62500000000000  E2
0.50000000000000   0.50000000000000   0.37500000000000  E3
0.50000000000000   0.50000000000000   0.87500000000000  E4
0.00000000000000   0.00000000000000   0.00000000000000  C
0.00000000000000   0.00000000000000   0.25000000000000  C
0.00000000000000   0.00000000000000   0.50000000000000  C
0.00000000000000   0.00000000000000   0.75000000000000  C

Quin_4 H1C0 HNF(n=4,2/7)=[1 0 0; 0 1 1; 0 0 4] site_config(1/24)=[0 0 0 0; 1 2 3 4] DG=8
1.000000
0.00000000000000   2.26569844629123   2.26569844629123
4.53139689258246   2.26569844629123   2.26569844629123
9.06279378516493   9.06279378516493   0.00000000000000
E1 E2 E3 E4 C
1 1 1 1 4
Direct(8) [A4B1C1D1E1]
0.50000000000000   0.50000000000000   0.00000000000000  E1
0.50000000000000   0.50000000000000   0.25000000000000  E2
0.50000000000000   0.50000000000000   0.50000000000000  E3
0.50000000000000   0.50000000000000   0.75000000000000  E4
0.00000000000000   0.00000000000000   0.00000000000000  C
0.00000000000000   0.00000000000000   0.25000000000000  C
0.00000000000000   0.00000000000000   0.50000000000000  C
0.00000000000000   0.00000000000000   0.75000000000000  C

Quin_5 H1C1 HNF(n=4,2/7)=[1 0 0; 0 1 1; 0 0 4] site_config(2/24)=[0 0 0 0; 1 2 4 3] DG=8
1.000000
0.00000000000000   2.26569844629123   2.26569844629123
4.53139689258246   2.26569844629123   2.26569844629123
9.06279378516493   9.06279378516493   0.00000000000000
E1 E2 E3 E4 C
1 1 1 1 4
Direct(8) [A4B1C1D1E1]
0.50000000000000   0.50000000000000   0.00000000000000  E1
0.50000000000000   0.50000000000000   0.25000000000000  E2
0.50000000000000   0.50000000000000   0.75000000000000  E3
0.50000000000000   0.50000000000000   0.50000000000000  E4
```

```
0.00000000000000   0.00000000000000   0.00000000000000  C
0.00000000000000   0.00000000000000   0.25000000000000  C
0.00000000000000   0.00000000000000   0.50000000000000  C
0.00000000000000   0.00000000000000   0.75000000000000  C

Quin_6 H1C2 HNF(n=4,2/7)=[1 0 0; 0 1 1; 0 0 4] site_config(3/24)=[0 0 0 0; 1 3 2 4] DG=8
1.000000
0.00000000000000   2.26569844629123   2.26569844629123
4.53139689258246   2.26569844629123   2.26569844629123
9.06279378516493   9.06279378516493   0.00000000000000
E1 E2 E3 E4 C
1 1 1 1 4
Direct(8) [A4B1C1D1E1]
0.50000000000000   0.50000000000000   0.00000000000000  E1
0.50000000000000   0.50000000000000   0.50000000000000  E2
0.50000000000000   0.50000000000000   0.25000000000000  E3
0.50000000000000   0.50000000000000   0.75000000000000  E4
0.00000000000000   0.00000000000000   0.00000000000000  C
0.00000000000000   0.00000000000000   0.25000000000000  C
0.00000000000000   0.00000000000000   0.50000000000000  C
0.00000000000000   0.00000000000000   0.75000000000000  C

Quin_7 H2C0 HNF(n=4,3/7)=[1 0 0; 0 1 2; 0 0 4] site_config(1/24)=[0 0 0 0; 1 2 3 4] DG=8
1.000000
0.00000000000000   2.26569844629123   2.26569844629123
6.79709533887370   4.53139689258246   2.26569844629123
9.06279378516493   9.06279378516493   0.00000000000000
E1 E2 E3 E4 C
1 1 1 1 4
Direct(8) [A4B1C1D1E1]
0.50000000000000   0.50000000000000   0.12500000000000  E1
0.50000000000000   0.50000000000000   0.37500000000000  E2
0.50000000000000   0.50000000000000   0.62500000000000  E3
0.50000000000000   0.50000000000000   0.87500000000000  E4
0.00000000000000   0.00000000000000   0.00000000000000  C
0.00000000000000   0.00000000000000   0.25000000000000  C
0.00000000000000   0.00000000000000   0.50000000000000  C
0.00000000000000   0.00000000000000   0.75000000000000  C

Quin_8 H2C1 HNF(n=4,3/7)=[1 0 0; 0 1 2; 0 0 4] site_config(2/24)=[0 0 0 0; 1 2 4 3] DG=8
1.000000
0.00000000000000   2.26569844629123   2.26569844629123
6.79709533887370   4.53139689258246   2.26569844629123
9.06279378516493   9.06279378516493   0.00000000000000
E1 E2 E3 E4 C
1 1 1 1 4
Direct(8) [A4B1C1D1E1]
0.50000000000000   0.50000000000000   0.12500000000000  E1
0.50000000000000   0.50000000000000   0.37500000000000  E2
0.50000000000000   0.50000000000000   0.87500000000000  E3
0.50000000000000   0.50000000000000   0.62500000000000  E4
0.00000000000000   0.00000000000000   0.00000000000000  C
0.00000000000000   0.00000000000000   0.25000000000000  C
0.00000000000000   0.00000000000000   0.50000000000000  C
0.00000000000000   0.00000000000000   0.75000000000000  C

Quin_9 H2C2 HNF(n=4,3/7)=[1 0 0; 0 1 2; 0 0 4] site_config(3/24)=[0 0 0 0; 1 3 2 4] DG=8
```

```
1.000000
0.00000000000000   2.26569844629123   2.26569844629123
6.79709533887370   4.53139689258246   2.26569844629123
9.06279378516493   9.06279378516493   0.00000000000000
E1 E2 E3 E4 C
1 1 1 1 4
Direct(8) [A4B1C1D1E1]
0.50000000000000   0.50000000000000   0.12500000000000  E1
0.50000000000000   0.50000000000000   0.62500000000000  E2
0.50000000000000   0.50000000000000   0.37500000000000  E3
0.50000000000000   0.50000000000000   0.87500000000000  E4
0.00000000000000   0.00000000000000   0.00000000000000  C
0.00000000000000   0.00000000000000   0.25000000000000  C
0.00000000000000   0.00000000000000   0.50000000000000  C
0.00000000000000   0.00000000000000   0.75000000000000  C
```

Supplementary Table 1: k-meshes of the structures generated by the AFLOW-POCC [2] algorithm.

Structure	k-mesh		
Ternary			
1	38	38	19
2	19	19	16
Quaternary			
1	38	38	13
2	19	19	10
3	19	19	16
Quinary			
1	19	19	5
2	19	19	5
3	19	19	5
4	19	19	8
5	19	19	8
6	19	19	8
7	19	19	12
8	19	19	12
9	19	19	12
10	19	19	17
11	19	19	17
12	19	19	17
13	19	19	12
14	19	19	12
15	19	10	10
16	19	10	10
17	19	10	10
18	19	16	16

2 SQS calculations

To achieve an equimolar ratio of all metals, we calculated and averaged 3 or 10 cells for the compositions with 3 or 5 metals, respectively, where the metals were permuted on the 11+11+10 or 7+7+6+6+6 sites. These supercells were relaxed with all degrees of freedom with a cutoff energy of 500 eV [1]. The stopping criterion for ionic relaxation was set to 10^{-4} eV. All SQS calculations were performed with a Γ-centered Monkhorst-Pack $6 \times 6 \times 6$ k-mesh [1], except for the binary carbides, which were calculated as single unit cells with a $12 \times 12 \times 12$ k-mesh. Below, 1 fully relaxed example of every structure is given.

2.1 Binaries

```
HfC
1.00000000000000
4.6487687022216235    0.0000000000000000    0.0000000000000000
0.0000000000000000    4.6487687022216235    0.0000000000000000
-0.0000000000000000   -0.0000000000000000    4.6487687022216235
Hf C
4 4
Direct
0.0000000000000000  0.0000000000000000 -0.0000000000000000
0.0000000000000000  0.5000000000000000  0.5000000000000000
0.5000000000000000  0.0000000000000000  0.5000000000000000
0.5000000000000000  0.5000000000000000 -0.0000000000000000
0.5000000000000000  0.5000000000000000  0.5000000000000000
0.5000000000000000  0.0000000000000000 -0.0000000000000000
-0.0000000000000000  0.5000000000000000  0.0000000000000000
0.0000000000000000 -0.0000000000000000  0.5000000000000000
```

2.2 Ternaries

```
1.00000000000000
9.0600066586398018   -0.0183441643964443    0.0042242025567306
-0.0183663343964022    9.0199429340781752   -0.0003305161665562
0.0042146292055332   -0.0003113338295406    9.0568616229783068
```

```
Hf Mo C
16 16 32
Direct
0.0007443253370256  0.4978100368341845  0.0013359462169287
0.0004960491145156  0.4990719603939502  0.4998815386701432
0.4945330985331589  0.0036876128411041  0.5036705507033681
0.4995233544382171  0.4998650682526837  0.0009602104497005
0.0001402663163092  0.7488629077056607  0.2486707899544269
0.4984900970886352  0.2465166691562705  0.7539243189474091
0.4985472253719728  0.7506174622630902  0.2462946703415706
0.4965456246825536  0.7551184539256454  0.7536336467687745
0.2528021649787439  0.4852776576941641  0.2555600808481967
0.2535102259252301  0.5036590222380334  0.7451047376860672
0.7469252166939226  0.9945183236401056  0.2538114647776825
0.7644584727105243  0.9999022631254123  0.7484464492113446
0.7465607961043373  0.5023018769624771  0.2536320891730321
0.7474045095152476  0.5006987137082217  0.7485185309882932
0.2478272080724716  0.7560157310759615  0.4954275327404967
0.7495288791618471  0.2496397677831163  0.5027083421483014
0.9800692013172553  0.0065122158855596  0.0165652094208035
0.0108581069115527  0.0108825119178246  0.4746966299870757
0.5289267370570669  0.0038760781295034  0.9997826316232422
0.5008879692358483  0.4994544723803713  0.4994996853651226
0.9738827991041530  0.2472946036849259  0.2435696210379586
0.0059133484374365  0.2698256173007892  0.7512872752836229
0.0034271759573132  0.7361348723533525  0.7515307804632080
0.5155953553477696  0.2448918633729433  0.2405929370975795
0.2597325500584371  0.0138584730744981  0.2229055255736953
0.2321465723513859  0.9937876958557091  0.7769427340718743
0.2438884352068304  0.2645500132000215  0.9827886608245728
0.2400565590767845  0.2399836419940739  0.5211138977664779
0.2422233987675807  0.7310560607760457  0.0025540985478716
0.7505949787979849  0.2593500920660020  0.0020816324802820
0.7604490050628279  0.7418765108262725  0.9854761723167017
0.7465643897170278  0.7465690788074578  0.5119886231622073
0.2496373378864964  0.0004744714614071  0.9998691419054975
0.2442898822724725  0.0059255820763499  0.5002759116840596
0.2488286508924238  0.4988245952020874  0.0002640144228368
0.2517987918926817  0.4953203887121929  0.4999662646953965
0.7520671422078276  0.0005241070514076  0.0008324965686326
0.7558373777677864  0.9969335403790511  0.5005500665028356
0.7502320754975934  0.4997096623511306 -0.0000112592524787
0.7481859220059474  0.5006944636049822  0.5007394742268453
0.0025457029318715  0.2446887852539832  0.0009013751776369
0.0030422275464173  0.2454125309944890  0.4998634435245535
0.0030106350167694  0.7555725608721676  0.9967345803696285
0.9972245671646945  0.7573076103474574  0.5031368871285102
0.4969621522339966  0.2438702685412701  0.0029546493560736
0.4934519834488147  0.2535749858049589  0.4977897324597364
0.4975346303004088  0.7581069684999178  0.9994806943965223
0.5013484895437096  0.7486853184096179  0.5002955306530852
0.0030323398688627  0.0036098488480153  0.2480750366877048
0.0097343941920065  0.0014966961433485  0.7535640204514451
0.9979557768488193  0.4952280308832041  0.2493785472796551
-0.0000063034053152  0.5009626735660265  0.7504798744413196
0.4970304861773576  0.0020646554456758  0.2450207088940149
0.5041925797258994  0.0007390559188865  0.7557273493896985
```

```
0.4997372324266434  0.4983185466444834  0.2510729202974626
0.5001298522911245  0.5008628672004540  0.7484568149073408
0.2500856523259671  0.2383706107362741  0.2485643037034673
0.2471176100803318  0.2457492584893875  0.7509029105261094
0.2500288651546733  0.7492741142118432  0.2450883138932804
0.2464177627795502  0.7547747962430500  0.7528885123201590
0.7467646580140594  0.2490824865393713  0.2498082472693073
0.7554511316220841  0.2505529068859252  0.7547875844907468
0.7498386001378922  0.7493870326150066  0.2503745635476648
0.7532376966981367  0.7504332508411587  0.7472102734331922
```

2.3 Quaternaries

```
Quaternary carbide Structure 1
1.00000000000000
9.0109202627730038   -0.0006598195908502    0.0010113700476912
-0.0006642699994660    9.0038861180669389   -0.0039747800179888
0.0010047569418917   -0.0039659491749780    9.0161270304712424
Hf Mo Nb C
10 11 11 32
Direct
0.0013852218666287  0.9996032378119044  0.4955915876336648
0.0035328070645370  0.4978047202501144  0.4998250397233047
0.5014650461782045  0.0011091341772746  0.9975220428360174
0.4969581377530676  0.4988830399696775  0.5018194111463683
0.9987705445455822  0.2458517364189401  0.2484099915705022
0.0063289628071843  0.2496682910247188  0.7519624598612643
0.4995784076910283  0.2539015524208553  0.2467087869650080
0.2478827988051133  0.9977328783940217  0.7502395074256948
0.7511098113927202  0.5032222649996311  0.2495679614617003
0.7534427003265520  0.2506268382582154  0.9987437801137384
0.0001446979690917  0.9894266553993813  0.0058235950084212
0.5019160357158496  0.5055680019758487  0.9950623560927319
0.0073213960299711  0.7491632804330564  0.2629527181899824
0.9907023592947996  0.7499486857085699  0.7314786520789074
0.2673103077225730  0.0021899128255800  0.2557935567466916
0.2459485350501942  0.5084547679101559  0.7665461703578362
0.7331303134118494  0.9959406555064556  0.2537020741526257
0.7586695384768899  0.5025830936425102  0.7520011416134141
0.2403985579737705  0.2287867535432009  0.0168197299180576
0.2530876450103688  0.2484969290424552  0.4816291368682789
0.2646616381841881  0.7467942917865789  0.9873743731175332
0.9976360865517008  0.5125186540926077  0.9977365886775923
0.4988870010627308  0.0033904456564116  0.5031363327814805
0.4904359191287500  0.2591581907677161  0.7556264195513815
0.4939687723411529  0.7490296357361010  0.2451439895299915
0.5040087762252267  0.7443475389675985  0.7546070654398223
0.2472570995194216  0.5070960593775600  0.2357754549477185
0.7539149473246529  0.0007070147976418  0.7496134170502404
0.2509606586784018  0.7452897354091197  0.5071857027879246
0.7480340656875685  0.2504204460614418  0.5037046092381715
0.7448167503813597  0.7506700799421380  0.0036249535033415
0.7481854022801292  0.7510008159039157  0.4961265056428455
0.2487067639013189  0.9923746955748957  0.0057931402225817
0.2521733866854068  0.9979828413782383  0.4991439914609769
```

```
0.2500364993049422  0.4969087333826661  0.9993143009236694
0.2500100638432156  0.4994465956413197  0.4992121462107870
0.7540409098988639  0.9969402500396776  0.0021772315327724
0.7481965405672968  0.9995981878484900  0.5009870335433275
0.7499791439061021  0.5038123890520253  0.9977323243994288
0.7505212348186495  0.5017520697634867  0.5020563983167645
0.0021492460610011  0.2491008027013320  0.0002161778722900
0.9990450141238878  0.2491905145041090  0.4996805540500545
0.0008465427390786  0.7510776798291801  0.9994511968198696
0.9997813062436138  0.7488414666182730  0.4986524279132207
0.4950401904566865  0.2538714136152239  0.9975123154422717
0.5002453480576521  0.2486020089256807  0.5018598254391160
0.5021950559724285  0.7508161945715018  0.0000169823493344
0.4999295064338665  0.7517046243138653  0.4998731486326330
0.0013035574415215  0.9938650771297146  0.2452783768445098
0.9987062444859830  0.9969662130002221  0.7509722346041073
0.0020663688255063  0.5008746424203819  0.2478068398701646
0.0013192623640130  0.5032172429761808  0.7508906539366140
0.5004311450616791  0.0002945799486227  0.2517314430653477
0.5010613093930204  0.0011757776982194  0.7475506397355973
0.4968195253262467  0.5045707977175075  0.2476240458888233
0.4996057127973226  0.5007382741373712  0.7516504519388764
0.2486049980123375  0.2494619138227390  0.2479428759104671
0.2529279817387413  0.2524642025141762  0.7541588315605708
0.2475034514830873  0.7530628236331411  0.2474426502618622
0.2461971206964741  0.7489722256967506  0.7503596672542099
0.7490118562963620  0.2482135709581546  0.2515408424613801
0.7494721487881907  0.2501807964942802  0.7496061943136354
0.7497260827031128  0.7543952735204713  0.2494066926559023
0.7504955391211248  0.7501407823606813  0.7505032525365849

Quaternary Carbide Structure 2
1.00000000000000
9.0275880239487805   -0.0048779345495507    0.0029985612582551
-0.0048645185728533    9.0105774994391847   -0.0003876771414007
0.0029806199299680   -0.0003957773215083    9.0337021792808887
Hf Mo Nb C
11 10 11 32
Direct
0.9991803495636083  0.5032548736400352  0.4978510962286553
0.0001738138186669  0.2464339026584017  0.2505270448375913
0.9972203666643642  0.2490641055064123  0.7506736381099886
0.5005504527733206  0.7496419828869567  0.2493612604349004
0.2560925884237055  0.0019608909544718  0.7486417567920136
0.7531672414572974  0.9994510835107907  0.2454311708262355
0.2520198412775077  0.2534367936191291  0.0014895851135813
0.2516915279183327  0.2483132334168116  0.4993074313264567
0.2511216938589504  0.7486758467643945  0.0017374208779626
0.7474226976680961  0.2470553481594116  0.9977255267945082
0.7480228095261017  0.2511682230396552  0.5001552247546207
0.0135945049563534  0.9952321641490687  0.9897165736658636
0.9845648703507675  0.9906526431007506  0.5170918417728644
0.4849005014732756  0.0067105198791004  0.0155960328014534
0.5084601930928178  0.5055540557045733  0.9848581399759742
0.9949929561261222  0.7492124461454143  0.2484015400905139
0.0191512719427020  0.7532019620746703  0.7552664181610020
0.4984043811651750  0.2445849311753563  0.2491074533872318
```

```
0.4850505755812398  0.7436200171299323  0.7513643323626819
0.7466778280355127  0.9853885252876406  0.7477164395843634
0.7564986239882029  0.7510936669583070  0.4835757056356426
0.9944880046826953  0.5037948843298177  0.0048863325959143
0.5084754838520542  0.9969230726392937  0.4947075363561855
0.5008237715212057  0.5059695639785580  0.5084986598310725
0.5051912893982700  0.2514792604799715  0.7500296115144285
0.2467734386944614  0.9983568762308580  0.2522826164404623
0.2551521168299062  0.4979283516367295  0.2520522479686402
0.2497948365743506  0.5005761771734687  0.7493633536728753
0.7450570582886296  0.4993656739603531  0.2486797841170393
0.7504445098853864  0.5121531993611643  0.7510185759329592
0.2468324210590472  0.7513526451204923  0.4992553206939648
0.7495790082375741  0.7523238854940838  0.0024420518424895
0.2491937938391013  0.0006214569297938  0.0017708904848209
0.2500108027170969  0.9965835681844317  0.4988533565353517
0.2505851029406302  0.5009219266931911  0.0002063282960324
0.2518936348399325  0.5012183072696542  0.5005059558460060
0.7484056416660506  0.9959814354564979  0.9927794803117775
0.7495789414673718  0.9978536549289149  0.5000190894870757
0.7505166761024670  0.5016628878925118  0.9993111437167802
0.7475144359903497  0.5040299907663799  0.4981263335092739
0.9997155957134027  0.2502762882362418  0.9998100912815151
0.9988048575256339  0.2474933048450579  0.5016410315487578
0.9988656664882558  0.7512267127184441  0.0001971642874425
0.9970658842529327  0.7527466631846892  0.5038753022370084
0.5003065899262074  0.2534583664095802  0.0003010753736072
0.5004969360004770  0.2506053529306845  0.5013478988624095
0.5025413290027240  0.7544141550834856  0.9958837314683523
0.5003631653307455  0.7501512144891493  0.5030077208887803
0.0015915117408656  0.9925433551016993  0.2516544624040188
0.9981757481385009  0.9967067924162135  0.7525891875168303
0.9993375224393655  0.5011187858119964  0.2485804537335688
0.0006690963134573  0.5056346961252161  0.7525452834947696
0.4988851118157983  0.9998661800863186  0.2519476079256634
0.5059492374416026  0.9984857878048440  0.7527298327495059
0.5000357433772034  0.4975591625354174  0.2497947516493679
0.5004379903812390  0.5005787558453076  0.7490826607716851
0.2509226880580606  0.2481286618633870  0.2500561128065130
0.2523205501949841  0.2526792259735687  0.7512207361480685
0.2463762844533132  0.7497515826507850  0.2529815683935874
0.2508735290995924  0.7501505313767728  0.7503031677996379
0.7475506450397562  0.2504882847911651  0.2482151283974851
0.7482691932173715  0.2514681246809065  0.7499384932018156
0.7503819656464236  0.7490866105252144  0.2453811124635610
0.7507931001534063  0.7525773702264119  0.7465311219087718

Quaternary Carbide Structure 3
1.00000000000000
9.0328594068906973    0.0053925176969893   -0.0005154571829415
0.0053877316226969    9.0186189534016350   -0.0011044705626729
-0.0005095222231505   -0.0011051214245671    9.0185890527608183
Hf Mo Nb C
11 11 10 32
Direct
0.0011752070344426  0.0008810883490246  0.0054910239178767
0.9992250922205793  0.4998132123838606  0.4990232289335066
```

0.4995224205950747	0.9993035832655530	0.0000364306661838
0.5003997718743821	0.9993894481542702	0.4973791529821991
0.5015237011426468	0.5015113098061864	0.4966190602455311
0.0001287641264944	0.7481257528236090	0.7551478555490982
0.4998003049463157	0.2482388016792713	0.7492080236160042
0.2462471303411382	0.4969592033687413	0.7531758876506013
0.7517463988198713	0.4995269247460767	0.7513566942479348
0.2475979603627136	0.2494408829115321	0.0001595021832133
0.2485744422313127	0.2477447456631605	0.4961232854358882
0.0006597347854173	0.0011398311909092	0.5104238763990701
0.9932935602462161	0.4945635951366374	0.0133258528524733
0.0174264668550677	0.2556352641968147	0.2485303296914108
0.9998866444974712	0.2370562165892022	0.7409320309932501
0.4829950299459487	0.2660377234879734	0.2467769624375585
0.5159788934381465	0.7378275712401136	0.2507936505191643
0.2724207614709667	0.9803835020039314	0.2483362058281825
0.2366691322008572	0.0012602894581043	0.7452358437145050
0.7303651857040683	0.0198011205088210	0.2473702128839693
0.7651986152697383	0.4771628332836224	0.2462370396737528
0.2537361902379114	0.7533449213494960	0.9896135940051574
0.5006191881363620	0.5037039985942927	0.0026712779052138
0.9874078618375478	0.7608382174772712	0.2530288036900025
0.4964887661900478	0.7518971464465535	0.7503241604706489
0.2414956411414886	0.5169267575724080	0.2467857885058222
0.7567701614875608	0.9984756433636159	0.7496156766729387
0.2460153907597307	0.7543359101408610	0.5086527885666928
0.7541527339542633	0.2504840087173855	0.9949581476098059
0.7544135203600851	0.2448443274882355	0.5026652360154378
0.7475992602943533	0.7496685390616370	0.0050155834786470
0.7506948462391883	0.7546662176340586	0.4964241309564248
0.2498868968181470	0.9977971193618942	0.9996168531005940
0.2462780523820971	0.9981394215606099	0.4941061068419561
0.2496321007221156	0.5033535561755836	0.0034195859733539
0.2502858222436436	0.5032375697202972	0.4968606046232563
0.7498360764146307	0.0006428225067597	0.0007995577788109
0.7525250268501256	0.0007613097060364	0.4955726334783304
0.7501374673863672	0.4990823936520062	0.0034976043733380
0.7505859301688639	0.4985758603099128	0.4961137282781709
0.9978730936720238	0.2503417669887172	0.9997948569805873
0.9989515083876143	0.2463421471037817	0.4959137089878271
-0.0000180740848024	0.7476898757651986	0.0067238544542701
0.9997088417787151	0.7526854005849994	0.5005247374296038
0.5012203559245861	0.2516539764932943	0.0015622835032113
0.5019856222010591	0.2504308871541008	0.4942361359468672
0.4989188095161557	0.7502336185741170	0.0011695120978481
0.4992335388768371	0.7508086311926044	0.5000236976571277
0.0006405068184356	0.0014779972450887	0.2582224436571181
0.9981427586531195	0.9967315057470778	0.7537789607363876
0.0007802258417153	0.5052131876158710	0.2512101890536151
0.9986509846411221	0.4948614164230380	0.7539616233040490
0.5012703352780102	0.0007498985652550	0.2485252732806389
0.5000183592824652	0.9971279914759827	0.7488274486154253
0.5040694422079638	0.4998717227776815	0.2475512719296356
0.4998941673388148	0.5039622351758287	0.7522873573248808
0.2514228199136016	0.2504377168958381	0.2480364936772009
0.2459036720161983	0.2466792228927970	0.7482414365922015
0.2502859054998957	0.7525591771938136	0.2485616591696778

```
0.2498175592093761  0.7503302485748795  0.7502477509913643
0.7524249979205696  0.2487534738096262  0.2483278462455576
0.7520964480902040  0.2476984752250017  0.7489728586772233
0.7504899252437703  0.7501271119869409  0.2504878049859092
0.7468220440391499  0.7506536734521515  0.7513867819557667
```

2.4 Quinaries

```
Quinary Carbide
1.00000000000000
8.9967916408865616   -0.0113783670500959   -0.0038146477733250
-0.0113553497868727    8.9969003047409544   -0.0033121261019237
-0.0038019280296021   -0.0033132903655984    9.0193622777090532
Hf Mo Nb Ta C
8 8 8 8 32
Direct
0.0024572023104454  0.0023014875200070  0.0032540141909585
0.4968207467773478  0.5017671486548945  0.0012761965079434
0.4996103643050889  0.5001417500131250  0.4995812853612493
0.9992647101789782  0.2491699900845208  0.7518125577188270
0.4912150772502122  0.2476240161616885  0.2504631624886755
0.2519558063745325  0.5061835489794260  0.7528436079283504
0.7520600111558964  0.2504175479088732  0.4973802847977361
0.7522353938289222  0.7504711006000575  0.4992990667939728
0.0010745102832415  0.5067163840218714  0.9850856237088376
0.0035851256756478  0.4960256389638545  0.5179147913885597
0.0070203767765999  0.2435468225518948  0.2506239109799764
0.5127656498390581  0.7551897014898493  0.7472922619002904
0.2486416331428654  0.9897039820413733  0.2480251389505430
0.2544937003844533  0.2440674869563721  0.9818647877346094
0.2481539200776045  0.2536326073658423  0.5183865470104781
0.2427350245380182  0.7559014051738207  0.0174205779517168
0.0068053513403377  0.0074495642232966  0.4921785838529109
0.4952884204969441  0.9989099553052729  0.5050153822083635
0.9951101279590441  0.7516912487136309  0.7494275313928341
0.4991264177287529  0.2474429008418481  0.7500463805445345
0.2516913438148013  0.9965258836799793  0.7490028250452341
0.2389424597837443  0.4965324872529672  0.2470577290760169
0.7469246653455025  0.9985258200700059  0.7498289618505796
0.7519172886342224  0.7470722045599921  0.9974577851341149
0.4961578609157269  0.9976284902605427  0.9963084080153429
0.0043532729816422  0.7521314163830245  0.2490679464712755
0.4954588434140899  0.7496224118834868  0.2498641254836091
0.7486101187564472  0.0022340812374794  0.2489469891019034
0.7587968471666358  0.4967628163709312  0.2474953664977064
0.7486624359309152  0.5007704388413640  0.7511676929806183
0.2479198504719865  0.7480280777322091  0.4940882282906019
0.7505021208602027  0.2529647011644289  0.0032207672135481
0.2516167490133780  0.0005314963349231  0.0006626847422322
0.2503146747887370  0.0002440087329915  0.4988784238649415
0.2460394373255243  0.5006300888472569  0.0037572868990740
0.2485070023264889  0.5009297853154795  0.4995890074544617
0.7468854408549684  0.0008275376852709  0.0005244137827836
0.7500912884662758  0.0000684415513560  0.4979338375341482
0.7509773217255894  0.4993065408574170  0.0000956330963514
```

```
0.7532446635444837  0.5005810816400149  0.4995375065912910
0.0008284607693747  0.2556121943002910  0.0035665709839505
0.0032266841904005  0.2530511321093143  0.4979782555367352
0.9993460423646671  0.7503807705849317  0.0004462602164133
0.0033253853684528  0.7508742680830283  0.4978598530943985
0.5025790918195967  0.2466175572961318  0.9988323383325686
0.4977885283033903  0.2474353482496571  0.5011970659812158
0.4983593619826354  0.7528939804765357  0.0006367411142339
0.4976841409955604  0.7522702451555484  0.4967220445454010
0.9983426048940298  0.9979953722407841  0.2498365433236300
0.0009694865150901  0.9984915870231920  0.7483081300096271
0.9992246018056183  0.4998068674009734  0.2490195527044274
0.9997503412259124  0.5016171737359377  0.7505893944626156
0.5003821772218261  0.9956623652787151  0.2470084705525133
0.4994455777885608  0.0004732495190799  0.7514595736225783
0.5001645254740938  0.5012322873202284  0.2492778930224060
0.5030802450155295  0.5001810458113203  0.7507901881970784
0.2437512341522902  0.2457266019139675  0.2506195487524525
0.2503493290969785  0.2503474944346912  0.7504234923943590
0.2495783235467109  0.7451290014612921  0.2544545989284779
0.2500133942159523  0.7549388399760130  0.7495237768794605
0.7504168358766592  0.2489526616755951  0.2479834866349535
0.7485941448054404  0.2514519919657543  0.7526320893683650
0.7507994911101553  0.7495218424509626  0.2477101376858704
0.7539667349157483  0.7490640235634373  0.7494466811190660
```

2.5 Hexinaries

```
Hexinary Carbide Structure 1
1.00000000000000
8.9364300217749584   -0.0017943438434327   -0.0001524556779338
-0.0017966283772354    8.9317966863278144   -0.0075379651565361
-0.0001506143218135   -0.0075292065682842    8.9339896573411721
Hf Mo Nb Ta Ti C
6 6 6 7 7 32
Direct
0.0003180498946371  0.9995681673651822  0.9978401282310778
0.5002987912947474  0.4994826955664452  0.9957965452153643
0.9992961618174454  0.7481085308477016  0.2515964166567257
0.2505226322299792  0.0004134435922157  0.7495547616261211
0.7494960792301167  0.2513659440867186  0.9988426545469170
0.7500393299872776  0.2511402080608808  0.5023283935251193
0.5049525803074033  0.5066471304644363  0.4994643870070575
0.5075287143535595  0.7524591875825797  0.7585408000131085
0.7454050959512347  0.9934564646999530  0.7488037399588698
0.7420709902325517  0.5078122176791872  0.2584168235617777
0.2484566725965628  0.2531458644419466  0.9949797051825344
0.7553672358210843  0.7468977252200116  0.5019825662145730
0.4997159812376368  0.0010151830979762  0.9943234918750479
0.0003431940794168  0.2524444029158486  0.7511476920799455
0.9933983219333620  0.7479597864752821  0.7465053658461722
0.4998556514256830  0.7495461682940393  0.2425301655421049
0.2541134055515770  0.4979145136014184  0.2508053709824775
0.2516591068731130  0.7480031759319075  0.9972598100681594
0.0003406308112175  0.4995824062768496  0.9965202916971381
```

```
0.5024878960161576  0.9948818887544749  0.5050349820455274
0.0023587255082888  0.2508782403333601  0.2512016769192797
0.4977967765258235  0.2490616577146142  0.2508874950849839
0.7497038911259669  0.9954117818593337  0.2500141064076587
0.2509407256080518  0.2531329847164562  0.5053674713804746
0.2452242381321947  0.7472776914468799  0.5013857153928015
0.9972059436243020  0.0004047729512634  0.5027902961024963
0.9979962185310131  0.4995068993402496  0.5035194989906622
0.5002865275609831  0.2525793826175547  0.7495230134574609
0.2499444983387949  0.0011013996895797  0.2509268871212882
0.2528868517775901  0.5009176272938252  0.7480553102203507
0.7471559624095062  0.5021954908742664  0.7461150964289039
0.7504568972948782  0.7475903425261824  0.9980865833163342
0.2527462317300996  0.9986949270197961  0.0044668782819697
0.2492511691157534  0.9995539783589201  0.4959412186097080
0.2471047753191377  0.5010037119521338  0.9949599210440635
0.2481405717419046  0.5006485361689357  0.5028159761901038
0.7477648630689130  0.9942982347735089  0.0014830079349071
0.7521753969399347  0.9967600347737626  0.4996891590651052
0.7535552482164577  0.5056469932061006  0.9987176084154664
0.7529699914260383  0.5030611723446379  0.5037062095632399
0.9989381296627549  0.2527226868133403  0.0025597339603212
0.0021411233298088  0.2501433348275809  0.4990168025790244
0.9973321578966549  0.7460670392570874  0.9979356042803712
0.0003822679115315  0.7495983804150025  0.5009843262661695
0.4993459426119460  0.2482008074149712  0.9994929605955553
0.4986799441948900  0.2482917740347960  0.5003901084261235
0.5051317889227298  0.7521119516351646 -0.0002845184266968
0.5008814868086663  0.7497253163078319  0.5006541896278351
0.0020701233715148  0.0027119477507932  0.2546315392312016
0.9980801561205105  0.9993475464276589  0.7444744251636919
0.0006504515971357  0.4969381323677818  0.2513614705926623
0.0006576140001918  0.5001508660871160  0.7490404509077921
0.4971707232202551  0.0008489057951430  0.2517478182655607
0.5003541414063670  0.0040566712640025  0.7488815325299314
0.4992004899480865  0.4994576386688745  0.2506468348068107
0.4996968491765691  0.5006391995774094  0.7452775909636914
0.2501694426406670  0.2468353284261700  0.2528079747948980
0.2531182462340859  0.2560393318784034  0.7477854498560412
0.2519227052264341  0.7520126070000044  0.2517986562199018
0.2507272501603979  0.7448850961497502  0.7469039102276515
0.7485322851703027  0.2485278335952305  0.2516468987397606
0.7475855627152836  0.2521666571769027  0.7496770890138214
0.7473798584155705  0.7507198219434790  0.2493602997994346
0.7485192336172363  0.7462301602690752  0.7512816297753964

Hexinary Carbide Structure 2
1.00000000000000
8.9389281937287048    0.0055545021599422   -0.0011119933121647
0.0055548865433166    8.9364332877885140    0.0060868276303801
-0.0011133703682112    0.0060858259558738    8.9290590996526618
Hf Mo Nb Ta Ti C
6 6 7 6 7 32
Direct
0.9995658212557123  0.4964370284784673  0.9991054950977802
0.9997607084922595  0.7520321717236808  0.2522179715720697
0.0001610999606199  0.7520682339956611  0.7482107277105138
```

0.2498584993447249	0.4986699226691427	0.2517777092249223
0.2524952704933515	0.7539478340521384	0.9990189794745900
0.7491166199099106	0.2476934511800064	0.5031886960034855
0.9941451798084572	0.4963814492496820	0.5026915273898560
0.4975006526576861	0.9971705694708268	0.5062357755122628
0.9947350099779199	0.2527770398023870	0.7420237150137083
0.7437095990862667	0.9952400219214179	0.7498704868138273
0.7550124843922832	0.5025981039939657	0.7547791961565280
0.2555795455561427	0.2458453746095089	0.9970322039688876
0.4976825838483255	0.0051263707611133	0.9946631681038929
0.0015554408802685	0.2477581814805227	0.2549381729715252
0.4965757868728620	0.2452471400471731	0.2465537377182214
0.5021307076200143	0.7539074242428947	0.7474374279306025
0.7497349753216057	0.0025673559247083	0.2489784469678287
0.7497010506436705	0.4972187200527601	0.2453685168360302
0.7512971191610200	0.7504531304870500	0.5023899139660590
0.0016007590860219	0.0051438453306473	0.9958977761044245
0.5013122556592351	0.4962687223542853	0.9964177546246294
0.5015599941608934	0.5008323493721885	0.5025685148935258
0.5004426113403341	0.7550804171696260	0.2528368907503880
0.2470315577205956	0.4951901010987666	0.7495688827800678
0.2500613584452313	0.2451633772134515	0.5042127189009457
0.0037910367273357	0.9998899276586940	0.5022681147084301
0.5028367950074831	0.2467591791691603	0.7525854488017809
0.2494470260801095	0.0018976512097039	0.2510980509165315
0.2515850631079030	0.0039874460179426	0.7491954475977356
0.2512327687885638	0.7546831449522873	0.5015510961393880
0.7490214241294127	0.2475366977388861	0.9958711725894714
0.7492911165347728	0.7532663111755586	0.9990207175082918
0.2481216379880669	0.0027142310825249	0.9994469996316874
0.2484698311343857	0.9989032948616234	0.5010478286328355
0.2545828748744100	0.4989029931923011	0.9972470576911411
0.2519559187951709	0.4997875059796222	0.5043019355021287
0.7509068706029748	0.0004431257716277	0.9993520518141922
0.7517484859709725	0.9972383229687243	0.4998382146526709
0.7471089016884366	0.4993139475958056	0.9996356150891272
0.7488125316826005	0.5003661487153839	0.4998025160670245
0.9963705354395469	0.2460857161796944	0.9980087381074580
0.0005668973211927	0.2457560542759887	0.5004718069665950
0.9945507926215393	0.7552613491853344	0.9997060265655427
0.0011304779837669	0.7532594470426309	0.5005427241381019
0.5053002181767167	0.2514553654671917	0.9949846059157497
0.4973380084654681	0.2504150520657814	0.5032142447827133
0.5066261178155049	0.7488889434721974	0.0002400848268090
0.4986650313322881	0.7490249103034331	0.5002908842648829
0.0024548004400185	0.0023235526301539	0.2489515196385682
0.0012899566442337	0.0055656884621049	0.7484919294518385
0.9983532016110266	0.4982203626852011	0.2525213611596777
0.0023055809485295	0.4986419467047765	0.7480857431617407
0.4959412400351354	0.9995319964880601	0.2503048411239026
0.4948737993924134	0.0039029172847948	0.7496110384388153
0.5008296426045979	0.5006339744115476	0.2496913896984875
0.4990502816412207	0.4976296000462380	0.7502532712101537
0.2501206099244356	0.2441531693335101	0.2533869017240062
0.2526283001607121	0.2475787147727797	0.7494880415862822
0.2538140370480779	0.7550520759577471	0.2562229763500180
0.2532428960550451	0.7506265057119385	0.7441195770356317

```
0.7494865413219381  0.2493930771605566  0.2458730178744121
0.7482887165919466  0.2492136581080662  0.7540476066965304
0.7470249371630615  0.7507273974734819  0.2486737974205360
0.7485084044535794  0.7501502600048875  0.7525711980325508

Hexinary Carbide Structure 3
1.00000000000000
8.9286664101408384    0.0016677820228710    0.0086062035393407
0.0016613537010452    8.9350498581536808    0.0039595628391399
0.0086155594851793    0.0039619867753221    8.9319774556223699
Hf Mo Nb Ta Ti C
6 7 6 6 7 32
Direct
0.0024806988746505  0.4996239246261815  0.5043986956285896
0.4977502452584579  0.4978402928318909  0.5035243828344623
0.4990948263449031  0.2473333091443066  0.2501854405958353
0.2503154407237014  0.9991459075106166  0.2501409550004173
0.7507322404511495  0.5005119034508237  0.2505736407418335
0.7501937035190129  0.2480065892443623  0.9981359219247150
0.9966755581612792  0.4922250058726381  0.9961881502468253
0.4912899398801345  0.0033748817975472  0.5099715999119383
0.5038189772481427  0.4976631114284284  0.9947886077562735
0.9868419315392473  0.7534803468295036  0.7498970630921211
0.2550892222370582  0.0217078929177012  0.7450069500295691
0.2509918414517048  0.4796842991671766  0.7629574879175682
0.2488735600863811  0.2425440263500461  0.4956155888123995
0.0039580847696314  0.0060370757339019  0.9943441553966677
0.0032203789435236  0.2482842303406797  0.2533705936056446
0.9986285506246286  0.7524822873727905  0.2504843368468371
0.5062463790215490  0.7527593647824942  0.7501886455366172
0.2492140000683785  0.5004005341273782  0.2407497166339989
0.7504522342048863  0.7533238840828647  0.9985960880436405
0.5007831888503271  0.7531690595783311  0.2494576682163681
0.7501193975327519  0.9972009344905205  0.7476612536204348
0.7518570602986065  0.5022437577909432  0.7500757613755027
0.2497161616333565  0.7496248166193729  0.9904356289579004
0.2484361037028336  0.7520092168208494  0.5089584289823801
0.7514307054691587  0.2434435831952920  0.5019676094399976
0.0022204559814019  0.9983619233957606  0.5036121649994627
0.4979649879614537  0.0042095679663940  0.9941063989110906
0.0039110496821377  0.2475977207942799  0.7471429124394222
0.4968224842786440  0.2499148119317857  0.7516900762729318
0.7501594924222400  0.9988849425035614  0.2523011095602122
0.2505647185932019  0.2522447791874528  0.0043400220887564
0.7500172873343464  0.7552467405788944  0.5004746605474784
0.2520386887461375  0.0003812867125744  0.9943974039734074
0.2448422146708999  0.9975078130031916  0.5029395083928869
0.2499091314271767  0.4995052068167058  0.9992201006145990
0.2489514377434598  0.5022745606170176  0.5022629098665116
0.7501047676534204  0.9977235385951987  0.9999971817896464
0.7510854526137457  0.9980673313747773  0.4989693764836572
0.7506802048725316  0.5014947909582504  0.9974653416476021
0.7509175285778473  0.5002703386461587  0.5041749760616375
0.0053128687757743  0.2491431690366304  0.9958097700637596
0.9985271678381012  0.2448123988778176  0.5034208150799373
0.0012852374510917  0.7512740198046397  0.9984003527543429
0.9979495668447510  0.7541389111155791  0.5012810706124136
```

```
0.4964066167191586  0.2479633394605051  0.9941233689075538
0.5011818431475177  0.2471099730668599  0.5064244438513481
0.4982017831288235  0.7534956011091405 -0.0000665479658005
0.4996329892343462  0.7532108326585445  0.5000855564021990
0.9955069814476241  0.9995397628435626  0.2512884317605825
0.0018465784314341  0.0019819770627844  0.7464309940000362
0.0023128886639400  0.4999129948894614  0.2500243947307136
0.0022567036791968  0.4972880543492226  0.7533790548951484
0.5046207729802246  0.9961123856339303  0.2490401920546374
0.5003504844465918  0.0048472558695285  0.7526813307082874
0.4978430364016203  0.5029029989196777  0.2488985307941021
0.4998073782408076  0.4970801045302039  0.7528254668372414
0.2494144397089251  0.2511536061349219  0.2456043501707922
0.2512603121840431  0.2505585195097935  0.7530630358382219
0.2516548350501705  0.7483114798801234  0.2484331673315490
0.2484435302905079  0.7515748271440790  0.7500942267996393
0.7516520623846111  0.2443351397761251  0.2526597321084164
0.7503788352354511  0.2495093456285933  0.7472709199427331
0.7485027750754178  0.7556497012080061  0.2513575369298297
0.7472499791857715  0.7502880123015945  0.7467012905944753

Hexinary Carbide Structure 4
1.00000000000000
8.9388961956518553    0.0022568171335227   -0.0013389232851347
0.0022568001303627    8.9420507869494887    0.0029458283673063
-0.0013404588822727    0.0029518579975363    8.9501171948505505
Hf Mo Nb Ta Ti C
7 6 6 6 7 32
Direct
0.9992785336512992  0.4976827711932476  0.9988795828071931
0.5017840222626375  0.9974350336882986  0.4983435181284535
0.9980714637564988  0.7476416219626199  0.7483015433282620
0.2504067066757694  0.9992917428947670  0.2508671007888764
0.7501649192544597  0.9997455646998027  0.7488708716357422
0.7498479208699996  0.2503690841771189  0.4986971292955531
0.7448661387319608  0.7480459074999503  0.0014853473066824
0.5071388389510506  0.5036755555138167  0.5025520961597473
0.0050926640466902  0.2537745792264868  0.7388306587064524
0.4990710238633908  0.7374764250714188  0.7440131288434373
0.2526970132932979  0.5050239023328854  0.7569982167305375
0.7489405878303238  0.0036240781470231  0.2458778372387063
0.2467601887954929  0.2557237665430967  0.5026278877298116
0.9933525167702149  0.4980505267187274  0.5012893486482894
0.5021674311639676  0.9972688691780301  0.0016452177125246
0.9961524795944467  0.2496527431175898  0.2564692063258063
0.5038881188262913  0.2523876570052568  0.2497607760299647
0.2535805106873776  0.2513147545237665  0.9985488105236372
0.7467979603643660  0.2495837090964910  0.0039767225112435
0.9981338417108380  0.0020337519077304  0.0003481697034632
0.9949064546921526  0.7501170465237967  0.2523174553363058
0.5013593529644279  0.2584593136156259  0.7499652887459227
0.2502981587145394  0.9963078543984498  0.7493470356112550
0.2541990083177124  0.5007028497981656  0.2461145042634612
0.2532566170883976  0.7449576086309921  0.5007958043591543
0.9987352889198200  0.9992103904308589  0.4988926123564168
0.5005022460843911  0.5024577495656399 -0.0000378953471515
0.5034060684634610  0.7472529275478639  0.2527887277425613
```

```
0.7460902613258316  0.5009758312092697  0.2506871410369345
0.7486985252164554  0.5002056589447339  0.7509191367672904
0.2544842429121785  0.7489806297839910  0.9986678442781816
0.7466462356255822  0.7491161301800345  0.4986009253249160
0.2491846887943375  0.9975032078662058  0.9968440741667738
0.2455378118158580  0.9969436305928392  0.5041474176192795
0.2546822100450346  0.5034085172104987  0.0017872453865067
0.2508615462134475  0.4998011232077739  0.5003909461553240
0.7510358292522442  0.0004608072420662  0.9993072659415394
0.7547440540732729  0.9952712624124632  0.4978755427158528
0.7453409560845357  0.4974378417980936  0.9998237318772613
0.7486513464482190  0.5053562429218939  0.5006485763133121
0.0007761537763319  0.2472041966831105  0.0000293459785832
0.0011920481513551  0.2465756816682318  0.4995532304572026
0.0031382254558152  0.7534698674359299  0.0048708511139016
0.9997253558260172  0.7513633159380791  0.4957823581394741
0.5002925393360347  0.2534584790157400  0.9993750212278807
0.4971293198494842  0.2531260392690702  0.5007576837176162
0.4943959264308025  0.7468898970135776  0.0015274990453615
0.5025186860691319  0.7466077560176210  0.4964529957565424
0.9988189272888003  0.9984695014912490  0.2516220791240190
0.0046398987263744  0.0026132003489245  0.7485333240462041
0.0005068258453240  0.5019085917099811  0.2522388761051609
0.9995164890700102  0.4971220778070345  0.7479080910133061
0.5011350096576890  0.9983144637079311  0.2477337782887221
0.4952583710835684  0.0024520284892773  0.7515040142927738
0.5003733472096208  0.5019468334822105  0.2494399833312255
0.5026648431747155  0.4999656305558459  0.7521928619633773
0.2501242963102825  0.2543333937321371  0.2502282318931582
0.2513675763329879  0.2511189956590391  0.7486040811436361
0.2505221216995011  0.7456156855072384  0.2504958469801851
0.2523505845760007  0.7503424986132107  0.7517779928455844
0.7490883979235723  0.2557987401864709  0.2489675428098097
0.7492705707421159  0.2558193572499957  0.7516605249456235
0.7488279159426694  0.7470210746793091  0.2552851461328814
0.7455527853694964  0.7437340253593969  0.7451920888422816

Hexinary Carbide Structure 5
1.00000000000000
8.9395056999045952    0.0031796320087475   -0.0006322020879768
0.0031702001462431    8.9499110199893508   -0.0018979569031427
-0.0006309103084542   -0.0018984952501704    8.9430879250997002
Hf Mo Nb Ta Ti C
6 6 7 7 6 32
Direct
0.0003550789766482  0.5025108033576745  0.4986636546060216
0.9988415333621056  0.2500082111469988  0.2493775034349269
0.5011290588121287  0.2503764491215574  0.2516901941332027
0.4960397527814420  0.7537930723253770  0.7529351899970340
0.7491008171622555  0.5007964707197874  0.7510525918637324
0.2481010987333891  0.2478182444231232  0.9978142066096231
0.0028230652152954  0.9976031415200337  0.5041438753852355
0.5040537962374990  0.0158222844155008  0.9980898100409226
0.4978292220788392  0.4853667767050335  0.9997094171391481
0.4983145198380532  0.7496492138544593  0.2480660926958231
0.2504313805799783  0.0044771695984438  0.7381753746688281
0.2499346517630848  0.7494744092075919  0.0072198912978380
```

```
0.0011096766325180  0.9937534992834174 -0.0018015266453438
0.9992614124893758  0.2457029892625266  0.7496276290198005
0.2550789726804983  0.5075798072593962  0.2438442033584953
0.2519144712068184  0.4983256101136336  0.7549028839682942
0.7453374835985767  0.9958700439657915  0.2478407253958163
0.7459225129638570  0.0000311136840730  0.7510711374893988
0.7512245446982532  0.2506811064136004  0.9982539846119922
0.5002864903250394  0.5046145446731893  0.4989292970153737
0.9987449435383327  0.7499733161156654  0.2524673137061865
0.0035970773001884  0.7531048888209293  0.7488608718356558
0.2539911217398589  0.9946865709966451  0.2548362925928541
0.2494588159715608  0.2465730017344449  0.5024000965179212
0.7511807847138384  0.2454764156643807  0.5009944755509047
0.7477067471133044  0.7477724373386058  0.0031115974449415
0.0022541844953631  0.5043308074341627  0.0012268148879842
0.5005807276415317  0.9961988448158706  0.4999439208038853
0.4994649455448539  0.2487294742047860  0.7477023007267314
0.7464345058763376  0.5027682881653401  0.2485976205721295
0.2512542449493214  0.7532368948239438  0.4992510263422855
0.7483391223356198  0.7542068492449595  0.4987074183352869
0.2526933881460154  0.9946149761789712  0.0000459649688976
0.2548856095574051  0.9973986580165967  0.4982555456654395
0.2475892265981985  0.5013309233975977 -0.0000840870816842
0.2536660583977584  0.5011856289393736  0.4997965827123448
0.7496046531875656  0.9984395345994532 -0.0000315008314304
0.7476004552139865  0.9970474030927157  0.4999575172290595
0.7513674460258757  0.5017514144088159  0.0051681550846362
0.7460928989877645  0.5017653030549845  0.4959593072511081
0.9979875925495563  0.2511824618884758  0.9978349486434448
0.0008038294779511  0.2492478302523259  0.5008541258705106
0.9990651502602327  0.7461187922648773  0.0004046618461697
0.0001379608792585  0.7517799887314984  0.5004349126391705
0.5007904320392532  0.2507094124858397  0.9941092297193023
0.4997623846358676  0.2492219848226739  0.5040350269250232
0.4995443998859729  0.7519293930955443  0.0048595416432991
0.4998589056214952  0.7511284298033829  0.4989900698042046
0.0009570835852288  0.9966479263301310  0.2494654714977004
0.9997325744513931  0.9987209537300724  0.7504897535866677
0.9983328287038448  0.5039573808002679  0.2453085820383299
0.0019949837926932  0.5022058955864682  0.7540970691821635
0.4993630126519892  0.9978031238544866  0.2515277197759449
0.4986014182108401  0.0058705900689228  0.7469260187698475
0.5031944721839466  0.5017595946705431  0.2504097056784280
0.4982607780001723  0.4974034007709687  0.7475553186281617
0.2502561381800728  0.2476616175921987  0.2529770628478318
0.2528504121602957  0.2504399036518296  0.7468355934431266
0.2490516698594154  0.7510853516651467  0.2548061935436732
0.2469321632926991  0.7510244669823167  0.7489750323660657
0.7497721157815211  0.2510634839484241  0.2516267905280758
0.7471076623883626  0.2487919142969459  0.7486473907446808
0.7500705276438036  0.7470648590145178  0.2523149116605543
0.7519750062937905  0.7523346515927317  0.7497414982163381

Hexinary Carbide Structure 6
1.00000000000000
8.9557899876465488   -0.0020504708029379   -0.0078950844486844
-0.0020578871142376    8.9306771591388507   -0.0013069068370678
```

```
-0.0079261397013203   -0.0012998558403663    8.9390713629220535
Hf Mo Nb Ta Ti C
6 7 6 7 6 32
Direct
0.9978964363506719  0.9943039792772604  0.4947494430879896
0.5019850624829022  0.9971244301868684  0.5028897998510934
0.0007919887422665  0.2507130954848254  0.7503419765076974
0.2525596581161111  0.9982506760557661  0.7512013354450028
0.2513241611274505  0.2500342182063330  0.0011146428415789
0.7497902663809272  0.2493941015226069  0.5008007600797925
0.5052854334394812  0.5023514139043962  0.5144496570649171
0.0011040639908931  0.7427333075976271  0.2470218552916716
0.4976571609743726  0.2547234366187392  0.7468959830819457
0.2699850586603006  0.9898132590595611  0.2497706200451005
0.2324221051381789  0.5124369316561360  0.2474575610139987
0.7312198585625800  0.0053396273206891  0.2444276274818112
0.7458289605410836  0.7440958087591764  0.5082593206053795
0.0003869130124442  0.9940797269709085  0.0054798088713596
0.0036655364000436  0.5052795148577242  0.0042644965662911
0.0039538712851172  0.2484107673418118  0.2507508237013303
0.0021358567272004  0.7481903761076197  0.7498613614715866
0.7601336790419609  0.4999703632015454  0.2512777582147472
0.2518769912918226  0.7450713487917747  0.4959518514548339
0.4968016574328593  0.5025076468725461  0.9919001137213238
0.4972614715044046  0.2551894499636856  0.2517992319656466
0.5043388687627024  0.7457786729435302  0.2492099803081713
0.7464849128335899  0.5010529610925601  0.7476042005642327
0.2518216828709161  0.2558722036928691  0.4996118489818694
0.7474343777516368  0.2456806097089939  0.0001033204645872
0.7502160068870977  0.7545478417492574  0.9953855968487908
0.9989699971456751  0.5025595524643643  0.4989935409007702
0.4995984571572615  0.9979414840578219  0.9975403735024567
0.4992383799461392  0.7513087015031942  0.7504410395527811
0.2531133395872500  0.5021171753264234  0.7487990267910870
0.7475595823270865 -0.0003096993301904  0.7516709244557446
0.2496740556492013  0.7504414794992122  0.0021605224876157
0.2534377420122710  0.9950155432114336  0.0029033421175687
0.2501756404734587  0.0012647764002090  0.4981602396293514
0.2523900191213312  0.5050961197284889  0.9966935947361737
0.2495884446674836  0.4996379386312739  0.5007542706558250
0.7467112695526558  0.9998958347449300  0.9960640914514137
0.7498811252279879  0.9952055058452117  0.5014821107963379
0.7488042838024898  0.5004636917971892  0.9992209076872868
0.7538249769572732  0.5008326340106443  0.5012097208015953
0.9962037185846195  0.2498431622674429  0.0037179825365142
0.0036520341852410  0.2526778553730640  0.4970204243254904
0.0014108118027407  0.7495013870673919  0.9994990420441199
0.9998483729045370  0.7434160678997868  0.4995983277718731
0.5021892532656730  0.2509267422402244  0.0019995801027665
0.4975518393650067  0.2531507984568513  0.4982184762344954
0.4998794527928572  0.7511425241099562  0.0003624958871238
0.4999880044512807  0.7462371997110907  0.5005897407919891
0.9991767233374337  0.9989848951930385  0.2467995528028974
0.9954808874177985  0.9954860820287029  0.7508654452371096
0.9983833287767109  0.4983469930432827  0.2554585049727129
0.9995428524075635  0.5031465279278637  0.7480201592018978
0.4998378644481676  0.9991388091685072  0.2461441768655711
```

```
0.5049176654663602  0.9973214574555535  0.7548330392053605
0.4989552573161289  0.5019296935657049  0.2499110052652862
0.4982332752252981  0.5065191868834775  0.7519589198863652
0.2499429302800759  0.2530283556687795  0.2524358201928718
0.2512239351321723  0.2556251389920775  0.7471161912565980
0.2511625699294984  0.7508367297196933  0.2447721887068817
0.2504179670726422  0.7441450223659349  0.7504852241714133
0.7492369649786573  0.2517826712320497  0.2471510543454274
0.7486002564336512  0.2501624223597689  0.7542159153692933
0.7500640051307939  0.7517727042952549  0.2486442994849900
0.7467706753585543  0.7504610661395048  0.7515077522422373

Hexinary Carbide Structure 7
1.00000000000000
8.9490108677905091    0.0028128622069270    0.0035651457816432
0.0028157702793764    8.9495829979619952    0.0031789186494968
0.0035665345537984    0.0031777067716935    8.9686523002714864
Hf Mo Nb Ta Ti C
7 6 6 7 6 32
Direct
0.0013923162086848  0.4973558714971054  0.4989202784753154
0.0005690542090534  0.7500537482720865  0.7518189075114959
0.2506692826235120  0.5018281857986897  0.2471798732928802
0.2499932440246986  0.4985848626727862  0.7525020219220545
0.7509377811998011  0.0011525089578009  0.7491560073998615
0.7495929880041670  0.5005829149012034  0.2485500803618581
0.7442644526309078  0.7482656804318926  0.0009530386790259
0.4929075555460412  0.4959436290094933  0.9957008285640871
0.0052224330876391  0.2439708576357502  0.2471011151457201
0.4989000369157904  0.7519623676677716  0.2706779714299877
0.4972603236392013  0.7551873889681653  0.7314157897109570
0.2532022555454099  0.9965507408385271  0.7570513264928455
0.2537196603848446  0.7491077335157500  0.9943229356916151
0.0064033159439745  0.0018098803717921  0.0017472134510021
0.0056730702641708  0.0035811300861279  0.4961985584131407
0.0044856331330084  0.7536716985554455  0.2464524383417148
0.4934598439403762  0.2484043353595473  0.7575242003316537
0.7515911212255438  0.0008220159008373  0.2507496482659325
0.2488880575526568  0.7549274607484372  0.5022716328054109
0.4942552693351918  0.9985918117139645  0.0010929926588004
0.4956825375902655  0.0024830621643003  0.4990160443222662
0.4983081882772351  0.4979874438508086  0.5029898522515404
0.4921771114223280  0.2518155404731172  0.2446799102622409
0.7486708386701444  0.4988302543857132  0.7522484891227015
0.7543685926794047  0.2511407583530886  0.0011954632997250
0.7542068975929073  0.2487073436403626  0.4987712576175324
0.0039325257330626  0.4971168199086964  0.0010991132186866
0.0055895339493258  0.2482905856766141  0.7524202939790261
0.2493666412343643  0.0005173630707035  0.2454451565563082
0.2447954203649594  0.2516797764071828  0.0011400694464913
0.2466686760006603  0.2467122658499136  0.4997643051429008
0.7540169525403888  0.7519304590839762  0.4989244444838774
0.2509707591944843  0.0020013466130540  0.0031598933872825
0.2521740858650970  0.0040287298042683  0.4969161095358632
0.2455305667622879  0.4965052846184574  0.0008271791271130
0.2529877728363034  0.4977193336852216  0.4989977553061096
0.7480169314888447  0.0024708872667868  0.0027614628518815
```

```
0.7489657112783595  0.0000215400294819  0.4971288219427225
0.7520168100775003  0.4964050278269396  0.9972194835128068
0.7475809789837560  0.5000474790281564  0.5024889560782905
0.0012777380898503  0.2510739990054003  0.9964932906008239
0.0017451351410284  0.2475339706488661  0.5030936092160194
0.0013795926075793  0.7486327552597740  0.0014324247141801
0.9992195556928698  0.7529608844969982  0.4986359143693092
0.4978107681135867  0.2473288461271315  0.0008461706586359
0.4988576776121980  0.2499942204982939  0.4997325597566787
0.4937202365636503  0.7506637414680588  0.9998348250549939
0.5050708507280118  0.7517851805857372  0.5005231283470837
0.0048233586325576  0.9994329832661839  0.2489366646409241
0.0039514998969053  0.0043398716131884  0.7501930803630074
0.0008478235791763  0.5005872195321521  0.2450545319559668
0.9954438845751306  0.4955509247711172  0.7535767268536885
0.4978710204715484  0.0009721343840419  0.2503041295409658
0.4986377227569146  0.9999125476503156  0.7502562299780546
0.4988423171639075  0.5014533047450298  0.2521780386734362
0.5009368865482933  0.5010743235708822  0.7489115057309369
0.2503450633041983  0.2458981106250664  0.2493436279263569
0.2479555323882564  0.2451317679523455  0.7510359372764922
0.2537061674108024  0.7549395538199200  0.2496524899628279
0.2527548717361703  0.7506300601699536  0.7500788324833364
0.7473708253831566  0.2485247102129809  0.2498233708041355
0.7511778785941425  0.2534054258335379  0.7506464736110395
0.7501643147364311  0.7530177248463887  0.2549183704985013
0.7466740503172811  0.7463896142765967  0.7459171465919012

Hexinary Carbide Structure 8
1.00000000000000
8.9434796081071894   -0.0041307888586347   -0.0021593103531749
-0.0041345633337353    8.9349864322186381    0.0003281934043443
-0.0021640325090039    0.0003249158239626    8.9363661135250290
Hf Mo Nb Ta Ti C
6 7 7 6 6 32
Direct
0.5009269110776876  0.9998390747222824  0.0028181933488091
0.4980165798689016  0.7460501352636002  0.2498160339170927
0.4944649039541111  0.7469918968802524  0.7497065154455911
0.7521695891155497  0.9979828703915847  0.7502455525834542
0.2512257686660794  0.2565629199253058  0.0037583441549484
0.7493622541518756  0.2531587387041390  0.4998262992466144
0.5112201804078158  0.4977711746509060  0.0072528410414496
0.9875293360520285  0.2511252490111622  0.2495672835274457
0.0080965855591517  0.7476131989364914  0.7507539341005951
0.5144038703681416  0.2557893931489392  0.2444058556721054
0.2568370495599492  0.4997651774797000  0.7380201662449870
0.7350470804602665  0.5013088408249914  0.7560488780101090
0.2422318943741544  0.7447768357063085  0.0021527862677168
0.0002897119966298  0.9970390503655714  0.4952109814754316
0.0051700494653280  0.5047837581321901  0.5041009152638588
0.5001013124777932  0.2547201235029229  0.7529635930923209
0.7523592823139755  0.5000535768473244  0.2477986983751263
0.2499927552708318  0.2546612543203024  0.4966126359303442
0.7530331037361803  0.7452465762089329  0.9977804034678148
0.7522985094881566  0.7456708568474979  0.5029839738875125
0.9997977505179541  0.9973063602037415  0.0049623851999843
```

```
0.9986724419158892  0.5029709604172541  0.9991772872699115
0.4996804574344999  0.0012336964668406  0.4962575318598219
0.9988583687030025  0.2529499352686966  0.7508996761943791
0.2472304669877234  0.0008561048831590  0.2510305031395057
0.2465580260757918  0.9993284433026781  0.7485054704246771
0.4957576472212415  0.5000682240583858  0.4938602432031380
0.0011517315570869  0.7469307894738532  0.2519497855149522
0.2469658120012971  0.5003574717933515  0.2556413305957488
0.7529468278603355  0.9955530688165362  0.2502408889274838
0.2480535858152209  0.7481775937138425  0.4958973344203655
0.7507630711789142  0.2508829148164037  0.0003266933591801
0.2466773781766512  0.0004926034401997  0.0001557226465154
0.2505273708508003  0.9994499181215003  0.4991961748032805
0.2498530402351204  0.5047593234057407  0.0018395088540248
0.2528709012320381  0.5026539593405034  0.4944282751585292
0.7543935258050279  0.0017143130647567  0.0051110418439770
0.7481281129773421  0.9988347491186312  0.4945055316895646
0.7527561226799877  0.4936549992588439  0.0008447192198068
0.7478369973854840  0.5021276954394317  0.5013122788194904
0.9957623887514180  0.2510127270843155  0.9992991354251722
0.0017057969048640  0.2517076962288777  0.5017558730810818
0.0003575247555192  0.7491304139669779  0.0031417635128987
0.0025802556974160  0.7495662887835941  0.4986422656773725
0.5059465412540010  0.2526935826742326  0.9990204029670511
0.4978432571314820  0.2522450582748010  0.4994902698097479
0.5001705344019962  0.7458266670714134  0.0001037521537708
0.4989463052442711  0.7499185825270865  0.4996952707648664
0.9986217211865417  0.9958330492814628  0.2488071416840769
0.0025107802028263  0.0011951921376760  0.7521964956518979
0.0027855189274114  0.5026297304205632  0.2498833181061769
0.0006412384291917  0.5006705698506732  0.7519172894305436
0.5003266033599906  0.0005915846110585  0.2529522353128000
0.4966148838542446  0.0017624742948343  0.7467294799719444
0.4975613658805819  0.4979950300918258  0.2519197394724593
0.4977756497001171  0.4991911803473523  0.7484228641715647
0.2493352790863400  0.2509728967330841  0.2528013462358686
0.2481418284928137  0.2499793271035690  0.7479685989547628
0.2447117486206951  0.7492273984599949  0.2508680399105147
0.2462763833723292  0.7499729959424976  0.7457730120323528
0.7506262477109749  0.2470161950426067  0.2450797357572470
0.7507802162415967  0.2520780198984600  0.7550343756819038
0.7544807267504910  0.7506015930242137  0.2509871970703972
0.7512408390668851  0.7469679198740671  0.7495441289658643

Hexinary Carbide Structure 9
1.00000000000000
8.9466763760037331   -0.0048684599755817   -0.0060261428830097
-0.0048762667158491    8.9546782002610001    0.0097050844293389
-0.0060318597590041    0.0096941992169560    8.9586150237801689
Hf Mo Nb Ta Ti C
7 6 7 6 6 32
Direct
0.0025791478835170  0.9980434082740437  0.0018809547086615
0.5015748051570383  0.5018558466721726  0.5018888424421545
0.0002131239796327  0.2509885435066235  0.7500902838048743
0.2509288138651608  0.4999503486842872  0.2495623729026397
0.7458610965358409  0.9957343929152188  0.2489860151033266
```

0.7468953062829576	0.2501650960674112	0.9972764313007135
0.7488077762885094	0.2508636580427598	0.5024020316859503
0.5002501008361159	0.4952613779624078	0.0003975922504514
0.9887024683042778	0.2535900028024106	0.2494446012787260
0.5094638868093603	0.2515759155281770	0.2463835183412355
0.2567189916447560	0.0043400834208332	0.2522833071081903
0.7495467955384144	0.5099835712487709	0.7502892651089667
0.2619979912957087	0.7504278006839603	0.9946831751675828
0.9985383463484133	0.5009309059179041	0.0000796239618752
0.4970740579611350	0.9996444404540413	0.4964078342472920
0.9961427472574339	0.7479145604939422	0.7511825795221565
0.2559447871813478	0.9970491183168437	0.7507868416438680
0.7439366040294774	0.9923015413329611	0.7491544173173409
0.2542663577421582	0.2478984895622114	0.0066639676172185
0.7383781174015370	0.7471936471996925	0.9946545153953825
0.0003205251296611	0.9995226043519685	0.4981357114514932
0.0020162087340707	0.7487191649542504	0.2494926118535093
0.2536584397809215	0.5011117897899663	0.7532009845942556
0.7496955548624863	0.5034013285557558	0.2520314898264010
0.2509742120969189	0.2476027169985632	0.4950016185421073
0.2521435745752328	0.7522018922303717	0.5023592217355453
0.9987566178929302	0.5022595661138907	0.5022200964106888
0.4973478600959405	0.0020436249976623	0.0010428966204361
0.5004122771000702	0.2492692244933590	0.7526538133410949
0.4972814085863104	0.7504958131857319	0.2474664612613647
0.5010287383043128	0.7501631284430753	0.7513282151218355
0.7479671238047534	0.7494065313671645	0.5027658683324510
0.2561319861071483	0.9988166228868777	0.0014036922074530
0.2488091764763583	0.0002423298216349	0.5001766876671239
0.2503768884329415	0.4995368008702198	0.9990595511205960
0.2457379938499520	0.4997255461717117	0.5027376024859098
0.7460235606205529	0.9967325651895319	0.9975355837464216
0.7511670850018912	0.9963928414041987	0.5005326658919068
0.7498983414824167	0.5010709674653147	0.0026097185895480
0.7547937701593337	0.5050095387423139	0.4978145042828400
0.0021436998526555	0.2515530550145992	0.0013259991799522
0.0026339488396125	0.2514487354351722	0.4990042913246251
0.9993145344533536	0.7472961615359506	0.9999017838163674
0.9991896380080453	0.7490710610558100	0.4997472274282467
0.4977986119436450	0.2465320169210943	0.9961311936304141
0.4968543241039481	0.2479171509998702	0.5029828271269471
0.4986249046774277	0.7515008549934159	0.9984631930237813
0.5005915722782235	0.7527975570518760	0.4999821342857348
0.0020833035706793	0.9980798457660141	0.2528711299142920
0.0010703770833949	0.9963525956839275	0.7473880115036512
0.9975266891792867	0.5031863909034296	0.2532977864736169
0.0027263475373008	0.5026621760355393	0.7477739014176409
0.4967449081467906	0.9969178636430488	0.2465274568458816
0.4992375639622251	0.9992079927494039	0.7517060127268139
0.5021001054462206	0.5048958384883341	0.2473163660512047
0.4990444764818924	0.5009212988321532	0.7536905935115207
0.2508887495571578	0.2488862815428741	0.2516284727235805
0.2558278238096929	0.2508463920475443	0.7497110895300177
0.2516581296899000	0.7534905125368691	0.2510288487376973
0.2536344634041151	0.7486220664495290	0.7489482042948707
0.7495642062034866	0.2526741856643424	0.2498752230710017
0.7444220561515322	0.2493814218686482	0.7496015669417408

```
0.7487413005277371  0.7460842262932718  0.2510825940244172
0.7452155996546804  0.7502369713670605  0.7459469264243745

Hexinary Carbide Structure 10
1.00000000000000
8.9469830329464699    0.0048644948241836   -0.0017772123493988
0.0048683435707988    8.9490317298522051    0.0035107854764769
-0.0017891562686708    0.0035089563047627    8.9495501583063284
Hf Mo Nb Ta Ti C
7 7 6 6 6 32
Direct
0.4985527754785268  0.5031839288750088  0.5033527110714008
0.0043867976317756  0.7509050613198682  0.7490567105101007
0.4979549253446290  0.2466320059467111  0.2487552892097883
0.7505021789851238  0.9990294529446093  0.7502050198490792
0.7518690774256511  0.2484883633443297  0.0016888655578260
0.7530205005575010  0.2488997713551064  0.4991279695153376
0.7489868654030810  0.7497861049695040  0.5008503213764302
0.0076839663597866  0.9967205613667136  0.4933779651342224
0.0039400235997155  0.2478636060428829  0.2542971850315456
0.4909121551471324  0.7622380313753890  0.7510977779190028
0.2535429836654777  0.9973507254924928  0.7591231209461418
0.7599635587660577  0.4984346985003524  0.7542505433851858
0.2576372971685872  0.7550198423036160  0.0131672454522856
0.2465326875766785  0.7543692272401454  0.4829347938802502
0.0048773129160212  0.0001269021387803  0.0034243717099506
0.0010084890053609  0.4993792631538629  0.0000131990388853
0.4918730505924573  0.9945177342755588  0.5011073776929955
0.0015614804564908  0.2508885108482257  0.7479558504042021
0.2468293341719591  0.4982443766469597  0.2499204764317976
0.7424053253658215  0.7492721573079756  0.9965775160053071
0.4974002467916492  0.5034022795996937  0.9971363856708817
-0.0005465710560171  0.7533227740494290  0.2495671889419219
0.4979041145161031  0.2433169801159508  0.7520046535823898
0.2455508666695938  0.5011962599525266  0.7496319202393613
0.7522513280157134  0.4989945436884077  0.2493316597392374
0.2459256020676434  0.2466563456652367  0.4990438144187545
0.9997247291498710  0.5032687670023365  0.4991179563916758
0.4971701098107766  0.9958860533913373  0.0013684833268724
0.5000811040035776  0.7533165618264221  0.2504660055335484
0.2516712982752739  0.0022056933468677  0.2427468568249485
0.7490544001314310  0.9992931910642023  0.2498180580313497
0.2491574153666156  0.2460995722036629 -0.0003692705466508
0.2546399173680868  0.0030032556330395  0.0031145128155219
0.2484560714422062  0.9997269511784879  0.4970960108372148
0.2509535733077774  0.4989037379175701  0.9993593878775243
0.2452759551502837  0.4989494741264243  0.5000023910153096
0.7498115308613887  0.9967234779483762  0.0062314403262755
0.7486941905245287  0.9995697652067126  0.4938664827363242
0.7493081262405265  0.5001784627091176  0.0013688131814869
0.7544356684954298  0.4988659759503339  0.4979659994804413
0.0061743835666180  0.2498078632177892  0.9997456412473099
0.0027540142073489  0.2522829082818012  0.5002293853649902
0.0001117790717418  0.7495956506493329  0.0019081425757605
0.0012177413543422  0.7471057126431321  0.4956998813965485
0.4957926214679874  0.2522140271299404  0.9961877109279704
0.4959365557328701  0.2490453518436576  0.5037034132711427
```

```
0.4988243629978883  0.7524738684510441  0.0025529031550021
0.4948010558079194  0.7515084325545852  0.4963839395142194
0.0006159268981333  0.9987923212358527  0.2500235247757379
0.0059592845900436  0.0012836854329230  0.7511720989641385
0.0002051576024783  0.5025970248312465  0.2520762245399006
0.0022874161721863  0.5001163107464274  0.7474275954246209
0.4987671941807271  0.9936431342226120  0.2483025215629126
0.4981282716231094  0.0012768208902396  0.7547646147440554
0.4994203040704397  0.5049690300522910  0.2468894778272299
0.4967486907979655  0.5003181106662230  0.7540612198664080
0.2485566015851846  0.2466404012708400  0.2488340773749154
0.2512304093379921  0.2501411615965500  0.7521956636176130
0.2511764884041272  0.7547604170249377  0.2486244079023075
0.2539009867297830  0.7507598919520400  0.7501261434977716
0.7507208421319195  0.2461084478796489  0.2503299410662278
0.7477397307507017  0.2504549094321088  0.7504530726100557
0.7498510336731125  0.7522243688399112  0.2475243918654949
0.7481186844950576  0.7476497011306369  0.7516309463615494
```

3 Plot data

The following tables contain the values of EFA (in $(\mathrm{eV/at})^{-1}$), σ_1 (in Å), and δ (in %) for all compositions shown in Figs. 1 and 2.

Supplementary Table 2: Plot data of investigated ternary compounds.

Material	EFA	σ_1	δ
HfMoC	5.5	0.0390	2.87
HfNbC	26.0	0.0218	1.77
HfTaC	18.6	0.0270	1.84
HfTiC	23.3	0.0425	3.37
HfVC	4.9	0.0995	5.43
HfWC	4.8	0.0421	2.81
HfZrC	166.8	0.0168	0.78
MoNbC	64.1	0.0465	1.10
MoTaC	84.0	0.0446	1.02
MoTiC	15.9	0.0236	0.51
MoVC	25.5	0.0505	2.56
MoWC	256.3	0.0473	0.06
MoZrC	4.6	0.0872	3.65
NbTaC	268.4	0.0120	0.08
NbTiC	2631.1	0.0248	1.61
NbVC	13.8	0.0640	3.66
NbWC	28.7	0.0510	1.04
NbZrC	12.9	0.0365	2.55
TaTiC	53.0	0.0294	1.53
TaVC	23.5	0.0596	3.58
TaWC	40.1	0.0376	0.97
TaZrC	9.7	0.0424	2.63
TiVC	18.1	0.0317	2.06
TiWC	34.0	0.0186	0.56
TiZrC	11.6	0.0569	4.16
VWC	105.1	0.0466	2.62
VZrC	3.7	0.1232	6.21
WZrC	3.9	0.0988	3.59

Supplementary Table 3: Plot data of investigated quaternary compounds.

Material	EFA	σ_1	δ	Material	EFA	σ_1	δ
HfMoNbC	13.4	0.0342	2.37	MoWZrC	7.7	0.1203	3.46
HfMoTaC	14.4	0.0417	2.38	NbTaTiC	25.9	0.0239	1.47
HfMoTiC	7.4	0.0526	3.00	NbTaVC	56.4	0.0380	3.38
HfMoVC	7.3	0.0996	4.44	NbTaWC	88.8	0.0261	0.95
HfMoWC	11.4	0.0835	2.70	NbTaZrC	13.2	0.0380	2.46
HfMoZrC	5.6	0.0385	3.11	NbTiVC	21.8	0.0438	2.99
HfNbTaC	19.5	0.0222	1.71	NbTiWC	14.5	0.0303	1.34
HfNbTiC	31.0	0.0321	2.76	NbTiZrC	17.6	0.0478	3.44
HfNbVC	7.0	0.0622	4.48	NbVWC	28.6	0.0560	3.06
HfNbWC	10.0	0.0365	2.33	NbVZrC	5.7	0.0820	5.07
HfNbZrC	24.3	0.0306	2.12	NbWZrC	7.6	0.0665	3.04
HfTaTiC	28.3	0.0340	2.76	TaTiVC	28.5	0.0435	2.93
HfTaVC	7.1	0.0635	4.47	TaTiWC	13.9	0.0303	1.27
HfTaWC	10.5	0.0363	2.34	TaTiZrC	16.7	0.0476	3.45
HfTaZrC	20.5	0.0350	2.19	TaVWC	24.4	0.0543	3.01
HfTiVC	8.5	0.0645	4.50	TaVZrC	5.6	0.0801	5.07
HfTiWC	7.5	0.0536	2.98	TaWZrC	7.7	0.0644	3.06
HfTiZrC	22.9	0.0420	3.57	TiVWC	11.9	0.0349	2.24
HfVWC	7.3	0.0938	4.44	TiVZrC	6.5	0.0848	5.22
HfVZrC	6.0	0.0727	5.42	TiWZrC	6.4	0.0753	3.73
HfWZrC	5.5	0.0448	3.06	VWZrC	5.5	0.1148	5.12
MoNbTaC	39.8	0.0314	1.00				
MoNbTiC	19.9	0.0297	1.35				
MoNbVC	49.9	0.0498	3.05				
MoNbWC	44.2	0.0556	1.02				
MoNbZrC	9.6	0.0574	3.08				
MoTaTiC	16.9	0.0279	1.28				
MoTaVC	41.4	0.0513	2.99				
MoTaWC	86.4	0.0508	0.94				
MoTaZrC	10.0	0.0635	3.10				
MoTiVC	17.2	0.0371	2.20				
MoTiWC	15.5	0.0424	0.50				
MoTiZrC	6.4	0.0784	3.75				
MoVWC	27.9	0.0469	2.42				
MoVZrC	5.8	0.1152	5.12				

Supplementary Table 4: Plot data of investigated quinary compounds.

Material	EFA	σ_1	δ	Material	EFA	σ_1	δ
HfMoNbTaC	7.0	0.0360	2.39	MoNbTaTiC	15.6	0.0354	1.57
HfMoNbTiC	6.1	0.0452	3.01	MoNbTaVC	15.5	0.0586	3.38
HfMoNbVC	5.4	0.0734	4.52	MoNbTaWC	16.8	0.0455	1.20
HfMoNbWC	5.9	0.0615	2.70	MoNbTaZrC	8.1	0.0495	3.14
HfMoNbZrC	6.3	0.0389	3.31	MoNbTiVC	11.8	0.0428	3.05
HfMoTaTiC	8.7	0.0436	3.00	MoNbTiWC	9.7	0.0362	1.36
HfMoTaVC	6.6	0.0710	4.51	MoNbTiZrC	7.6	0.0601	3.75
HfMoTaWC	5.6	0.0624	2.70	MoNbVWC	13.2	0.0792	3.09
HfMoTaZrC	5.9	0.0468	3.34	MoNbVZrC	5.1	0.0792	5.15
HfMoTiVC	6.2	0.0628	4.50	MoNbWZrC	4.9	0.0951	3.46
HfMoTiWC	4.9	0.0689	3.10	MoTaTiVC	11.3	0.0410	2.99
HfMoTiZrC	6.0	0.0537	4.13	MoTaTiWC	9.1	0.0296	1.28
HfMoVWC	5.3	0.0949	4.44	MoTaTiZrC	7.6	0.0572	3.75
HfMoVZrC	3.8	0.0875	5.63	MoTaVWC	12.9	0.0552	3.04
HfMoWZrC	3.4	0.0626	3.79	MoTaVZrC	5.1	0.0757	5.14
HfNbTaTiC	20.6	0.0338	2.76	MoTaWZrC	5.0	0.0795	3.47
HfNbTaVC	9.7	0.0477	4.50	MoTiVWC	9.0	0.0454	2.44
HfNbTaWC	4.3	0.0390	2.36	MoTiVZrC	4.8	0.1013	5.23
HfNbTaZrC	7.8	0.0367	2.62	MoTiWZrC	4.3	0.1017	3.90
HfNbTiVC	9.2	0.0554	4.62	MoVWZrC	4.4	0.1285	5.15
HfNbTiWC	7.4	0.0454	2.99	NbTaTiVC	26.6	0.0384	3.41
HfNbTiZrC	13.6	0.0388	3.70	NbTaTiWC	11.8	0.0327	1.55
HfNbVWC	5.9	0.0694	4.51	NbTaTiZrC	12.6	0.0487	3.47
HfNbVZrC	5.6	0.0607	5.44	NbTaVWC	14.0	0.0456	3.38
HfNbWZrC	5.3	0.0471	3.27	NbTaVZrC	6.8	0.0598	5.07
HfTaTiVC	10.1	0.0528	4.60	NbTaWZrC	6.6	0.0503	3.11
HfTaTiWC	7.3	0.0485	2.98	NbTiVWC	9.9	0.0417	3.06
HfTaTiZrC	10.8	0.0450	3.72	NbTiVZrC	6.5	0.0694	5.26
HfTaVWC	5.2	0.0692	4.50	NbTiWZrC	5.9	0.0592	3.73
HfTaVZrC	5.4	0.0631	5.44	NbVWZrC	4.8	0.0852	5.14
HfTaWZrC	5.0	0.0417	3.30	TaTiVWC	9.3	0.0359	3.01
HfTiVWC	6.0	0.0749	4.50	TaTiVZrC	6.9	0.0684	5.25
HfTiVZrC	5.4	0.0721	5.79	TaTiWZrC	6.0	0.0568	3.73
HfTiWZrC	5.1	0.0599	4.10	TaVWZrC	4.9	0.0718	5.13
HfVWZrC	3.6	0.0949	5.62	TiVWZrC	5.2	0.1036	5.23

Supplementary Table 5: Plot data of investigated hexinary compounds. * taken from [3].

Material	EFA*	σ_1	δ	Material	EFA*	σ_1	δ
HfMoNbTaTiC	83	0.0406	3.01	MoNbTaTiVC	100	0.0365	3.41
HfMoNbTaVC	71	0.0506	4.56	MoNbTaTiWC	77	0.0345	1.62
HfMoNbTaWC	71	0.0490	2.70	MoNbTaTiZrC	71	0.0504	3.75
HfMoNbTaZrC	71	0.0436	3.45	MoNbTaVWC	125	0.0503	3.38
HfMoNbTiVC	71	0.0541	4.63	MoNbTaVZrC	59	0.0690	5.16
HfMoNbTiWC	53	0.0497	3.14	MoNbTaWZrC	63	0.0670	3.47
HfMoNbTiZrC	67	0.0469	4.16	MoNbTiVWC	71	0.0447	3.09
HfMoNbVWC	56	0.0722	4.54	MoNbTiVZrC	59	0.0724	5.30
HfMoNbVZrC	50	0.0674	5.63	MoNbTiWZrC	48	0.0692	3.91
HfMoNbWZrC	48	0.0573	3.84	MoNbVWZrC	48	0.0939	5.19
HfMoTaTiVC	67	0.0509	4.61	MoTaTiVWC	67	0.0442	3.04
HfMoTaTiWC	53	0.0487	3.12	MoTaTiVZrC	59	0.0699	5.28
HfMoTaTiZrC	63	0.0483	4.17	MoTaTiWZrC	48	0.0668	3.91
HfMoTaVWC	56	0.0697	4.53	MoTaVWZrC	48	0.0899	5.18
HfMoTaVZrC	50	0.0645	5.63	MoTiVWZrC	40	0.0857	5.24
HfMoTaWZrC	45	0.0643	3.86	NbTaTiVWC	77	0.0357	3.41
HfMoTiVWC	45	0.0706	4.50	NbTaTiVZrC	83	0.0571	5.28
HfMoTiVZrC	50	0.0754	5.89	NbTaTiWZrC	59	0.0523	3.73
HfMoTiWZrC	38	0.0709	4.42	NbTaVWZrC	56	0.0717	5.15
HfMoVWZrC	37	0.1029	5.75	NbTiVWZrC	50	0.0718	5.29
HfNbTaTiVC	100	0.0451	4.67	TaTiVWZrC	50	0.0703	5.28
HfNbTaTiWC	67	0.0402	2.99				
HfNbTaTiZrC	100	0.0403	3.80				
HfNbTaVWC	67	0.0556	4.55				
HfNbTaVZrC	67	0.0536	5.45				
HfNbTaWZrC	59	0.0457	3.41				
HfNbTiVWC	59	0.0546	4.63				
HfNbTiVZrC	71	0.0606	5.79				
HfNbTiWZrC	53	0.0522	4.13				
HfNbVWZrC	45	0.0721	5.62				
HfTaTiVWC	56	0.0537	4.61				
HfTaTiVZrC	71	0.0603	5.79				
HfTaTiWZrC	50	0.0539	4.14				
HfTaVWZrC	43	0.0739	5.62				
HfTiVWZrC	45	0.0798	5.88				

References

[1] J. D. Pack, H. J. Monkhorst, Special points for Brillouin-zone integrations, Physical Review B 16 (4) (1977) 1748–1749. doi:10.1103/PhysRevB.16.1748.

[2] K. Yang, C. Oses, S. Curtarolo, Modeling off-stoichiometry materials with a high-throughput ab-initio approach, Chemistry of Materials 28 (18) (2016) 6484–6492. doi:10.1021/acs.chemmater.6b01449.

[3] P. Sarker, T. Harrington, C. Toher, C. Oses, M. Samiee, J.-P. Maria, D. W. Brenner, K. S. Vecchio, S. Curtarolo, High-entropy high-hardness metal carbides discovered by entropy descriptors, Nature Communications 9 (1) (2018) 4980. doi:10.1038/s41467-018-07160-7.
